# Online Gambling Detection Systems: A Systematic Review

**DOI:** 10.12688/f1000research.191912.2

**Published:** 2026-11-11

**Authors:** Abdul Azzam Ajhari, Rizal Fathoni Aji, Aprinaldi Jasa Mantau, Mahardhika Pratama, Wisnu Jatmiko

**Affiliations:** 1 Universitas Indonesia Fakultas Ilmu Komputer, Depok, West Java, Indonesia; 2 National Cyber and Crypto Agency, Depok, West Java, Indonesia; 3 STEM, University of South Australia, Adelaide, South Australia, Australia

**Keywords:** systematic review, online gambling detection systems, website detection, mobile app classification, social media

## Abstract

**Background:**

Illegal online gambling increasingly exploits websites, mobile applications, social media, messaging services, and decentralized financial infrastructure, and operators actively evade automated filtering. This review synthesizes how online gambling detection systems (OGDS) are applied, integrated, and evaluated, and which limitations constrain their deployment.

**Methods:**

We searched IEEE Xplore, ACM Digital Library, ScienceDirect, and Scopus on 9 January 2026. Peer-reviewed English-language journal and conference studies in computer science published from 2021 to 2025 and reporting measurable evaluation metrics were eligible. We appraised methodological quality using a 10-item checklist and quantified inter-reviewer agreement with Cohen’s kappa. We synthesized evidence through frequency analysis and thematic synthesis across four research questions.

**Results:**

Of 1,866 records identified, we included 39 studies (kappa = 0.83). Website detection dominated the corpus (15 studies), followed by harm detection (9), social media and spam messages (6), crypto and blockchain (4), mobile applications (3), and network infrastructure (2). Researchers identified six architectural integration patterns: multimodal feature fusion, graph-structural integration, hybrid ensemble learning, transformer-based semantic systems, harm systems, and rule-based operational systems. Multimodal fusion and graph-based models reported the highest detection scores against content obfuscation, whereas lightweight rule-based tools were favored for low-resource infrastructure. Evidence quality was uneven: 12 studies lacked a baseline comparison, 27 did not release data or code, and only six reported computational cost; differing datasets and protocols preclude direct performance comparison. Four recurrent limitations emerged: data quality and temporal drift; adversarial evasion and multimodal obfuscation; semantic ambiguity of localized slang and blockchain anonymity; and computational constraints.

**Conclusions:**

Research priorities include adaptive recalibration, lightweight edge deployment, deep semantic entity extraction, explainable models for legal admissibility, and Web3 forensics. Shared, longitudinal, and cross-regional benchmarks are needed before performance claims can be generalized beyond individual study settings.

## Introduction

The rise of online gambling activities has become a serious threat to the integrity of the digital infrastructure, where more than 99.5% of blocked domains in Indonesia are related to gambling. ^1^ These attacks target not only the commercial sector but also university domains and government websites through web defacement techniques and hidden-page infiltration. ^2^ ^,^ ^3^ In social media, illegal content spreads rapidly, with more than 12 million promotional posts discovered on Twitter within six months. ^4^ The sheer scale of this threat, coupled with the involvement of organized criminal syndicates, demands a more responsive and intelligent automated detection system. ^5^ ^,^ ^6^ Case studies from Taiwan show that online gambling is frequently combined with organized crime, fraud, and money laundering, and that operators adopt new technologies to conceal their activities and circumvent police investigations. ^7^ ^,^ ^8^ Offshore operators also exploit jurisdictional gaps between countries in which gambling is prohibited or restricted, ^9^ and mobile gambling scams have been linked to money-mule payment channels with average daily gambling deposits of about USD 400,000. ^10^

Online gambling is also a growing public-health concern. Online-only and mixed-mode gamblers differ from land-based gamblers in demographic and harm profiles, ^11^ and problematic online gambling forms a related but distinct condition within the spectrum of problematic online behaviors. ^12^ Social media cliques, perceived group norms, and online communities are associated with young people’s interest in gambling content and with problem gambling, ^13^ ^–^ ^15^ and application-based gambling advertising has been linked to youth willingness to gamble. ^16^ Gambling-like mechanics are spreading into adjacent digital products: loot box purchasing is associated with problem gambling and gaming disorders, ^17^ ^–^ ^20^ gacha monetization raises similar concerns, ^21^ only about 7% of loot box advertisements on Meta and TikTok disclosed their presence, ^22^ user-generated game platforms expose children to gambling-like gameplay, ^23^ and gamblification and esports betting extend gambling logics to new media. ^24^ ^,^ ^25^ Researchers have used bet-level data from 36,328 online sports bettors to examine whether early big wins predict future involvement, ^26^ and digital payment solutions have raised concerns about facilitating harmful gambling. ^27^ Harm-reduction technologies, including online self-exclusion services, ^28^ ^–^ ^30^ internet-delivered psychological interventions, ^31^ family-oriented collaborative systems, ^32^ and explainable persuasive interfaces, ^33^ ^–^ ^35^ complement but do not replace efforts to identify illegal gambling services. In Indonesia, sentiment analysis shows predominantly negative public sentiment toward online gambling, ^36^ and survey research has examined the factors behind gamblers’ intentions to discontinue. ^37^ Computing research on gambling further includes public perceptions of casino legalization, ^38^ usability benchmarking of electronic gambling equipment, ^39^ and studies of the betting market itself, such as odds and team-strength evaluation in football, ^40^ skill-versus-chance quantification in daily fantasy sports, ^41^ simulators of in-play betting behavior, ^42^ and the engineering of online betting games. ^43^ These strands do not address the automated detection of illegal gambling services, which is the focus of this review.

The evolution of online gambling detection systems (OGDS) began with user behavior analysis that focused on identifying addiction and harm patterns through banking transaction data and time series of betting activities. ^44^ ^,^ ^45^ However, these behavior-based methods often fail to detect new platforms in real time because of the limited access to private data and dynamic variations in user behavior. ^46^ Subsequently, the architecture shifted toward text and HTML content analysis. Still, perpetrators began using keyword obfuscation techniques, slang such as “ *gacor*,” and image-based text to evade purely text-based filters. ^47^ ^–^ ^49^ This led to the emergence of a multimodal architecture that integrates text, visual (screenshot) features, and encrypted traffic metadata to overcome the detection weaknesses of a single modality. ^50^ ^,^ ^51^

Modern detection systems currently adopt a hybrid approach that combines automated web crawling with advanced deep learning models. Heterogeneous graph neural networks (HGNNs) and user interface (UI) transition graphs (UTGs) allow the system to recognize the structure of gambling applications or websites, even when their content is disguised as normal sites (fake-normal). ^52^ ^,^ ^53^ In addition, integrating large language models (LLMs) such as BERT (including IndoBERT) has been shown to improve promotion-detection accuracy on platforms such as YouTube and Twitter by capturing complex semantic contexts. ^4^ ^,^ ^54^

The literature reports substantially higher efficiency for automated methods than for traditional manual approaches. Table 1 compares the main architectural approaches reported in the literature.

**Table 1.** Architectural comparison of online gambling detection system approaches.

Aspect	Traditional	Behavior-based	Content-based	Graph-based	Multimodal
**Speed and efficiency**	Manual forensic workflows exhibit high latency due to human-in-the-loop verification ^55^	Near real-time risk scoring achieved via in-database feature extraction pipelines ^56^	Fast online detection enabled by lightweight lexical pipelines for text features ^47^ ^,^ ^48^ ^,^ ^57^	Efficient large-scale inference using similarity graph embeddings ^58^	Higher inference latency due to cross-modal fusion and vision–language attention mechanisms ^49^ ^,^ ^59^
**Detection scope**	Limited to predefined attack signatures and static URL patterns ^2^	Focuses on identifying potentially problematic gambling profiles through longitudinal betting trajectories ^44^	Targets semantic patterns of gambling promotion in user-generated content ^54^	Captures fake-normal websites by modeling UI transitions and hyperlink relationships ^52^	Covers concealed promotions embedded in screenshots and dynamically rendered web pages ^50^
**Scalability**	Scalability constrained by manual inspection and static blacklist maintenance ^60^	Scales within regulated financial environments but constrained by data availability ^45^	Highly scalable crawling and indexing of social media platforms ^4^	Designed to scale across underground ecosystems such as Telegram-based app distribution ^61^	Scalability enhanced through multimodal data augmentation and semi-supervised learning ^62^
**Robustness**	Easily bypassed by minor keyword variations and dynamic hosting infrastructures ^60^	Sensitive to temporal drift as gambling behaviors evolve over time ^63^	Vulnerable to semantic obfuscation and slang evolution in promotional content ^47^	Robust against content evasion through UI-transition correlation and traffic analysis ^51^	Most resilient by cross-validating semantic, visual, and structural inconsistencies ^59^

UI, user interface; URL, uniform resource locator. Numbers in brackets refer to the reference list.

Traditional (manual or rule-based) methods are the most basic approaches that rely heavily on human intervention or static rules. Manual methods, such as search engines, have proven inefficient because they often return inactive results or are blocked by firewalls. ^60^ Furthermore, manual analysis using simple tools such as spreadsheets is considered error-prone when handling large volumes of investigative data. ^55^

Behavior-based methods focus on identifying gambling activities using user interaction patterns and transaction logs. This approach can map user profiles from occasional players to players with potentially problematic gambling patterns by analyzing the time series of the betting activity. ^44^ Using payment data also enables identification of risk indicators, such as a high frequency of deposits relative to withdrawals. ^45^ However, the main challenge is data drift: user behavior patterns change over time, which can reduce the detection model's reliability if it is not updated regularly. ^63^

Content-based methods scan HTML text, social media comments, or spam SMS messages to identify gambling promotion keywords. Advanced language models such as IndoBERT effectively understand the context of promotions in local languages, including certain slang terms. ^47^ ^,^ ^54^ However, this method often faces evasion tactics, in which perpetrators disguise links or use words that standard text filters cannot read. ^48^ ^,^ ^57^

Hybrid/graph-based methods go beyond text analysis by mapping the structural relationships between applications and websites. By analyzing hyperlink relationships ^52^ and UTGs, ^58^ the system can identify websites and apps whose surface content appears harmless to conventional detectors. Furthermore, analyzing encrypted traffic and server metadata allows detection of gambling apps, even when rapid domain switching hides their true identity. ^51^

Multimodal methods are the most comprehensive architectures because they combine visual, textual, and metadata features simultaneously. By integrating screenshots (visual) and OCR-extracted text, this system can still detect illegal content even when the perpetrator hides promotional keywords in the image to avoid HTML-based sensors. ^49^ ^,^ ^50^ This approach is less vulnerable to single-channel evasion because it cross-verifies multiple data modalities, reducing detection errors that may occur when relying on a single feature type. ^59^ ^,^ ^62^

Figure 1 provides a comparative overview of the five foundational OGDS architectures to clarify the evolution and rationale behind modern hybrid systems. As shown in Figure 1a, traditional methods operate within a manual forensic framework, where human-in-the-loop verification filters search engine results. Although accurate in specific cases, this linear workflow has high latency and is easily bypassed by dynamic hosting infrastructure. By contrast, Figure 1b presents behavior-based detection, which shifts the focus from the website itself to the user’s longitudinal transaction trajectories. This enables the identification of potentially problematic gambling patterns, even on seemingly normal platforms; however, it remains constrained by the limited availability of private financial data and sensitivity to temporal drift.

**Figure 1.**
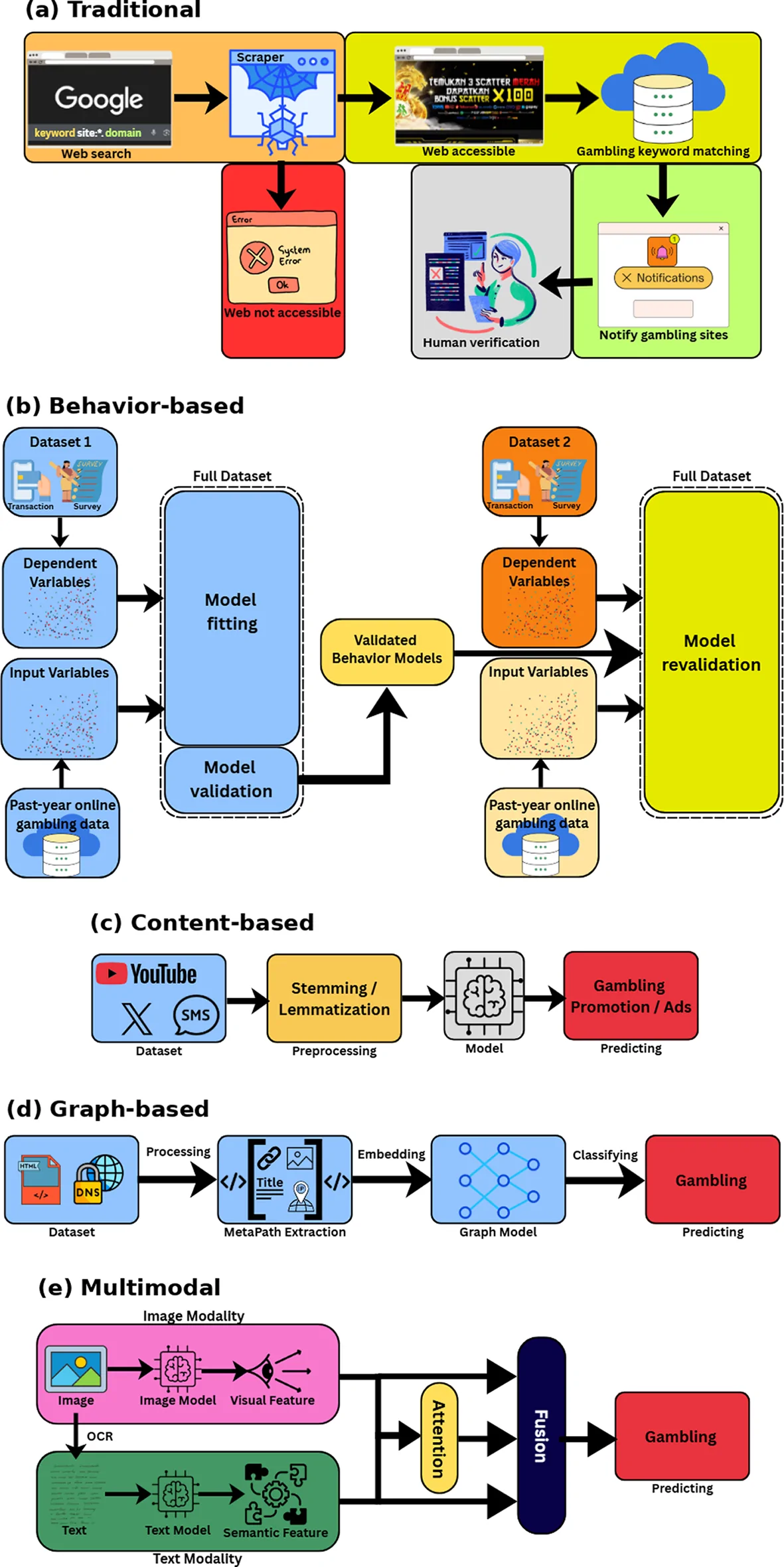
Five foundational architectures of online gambling detection systems. (a) Traditional manual forensic workflow with human-in-the-loop verification of search-engine results; (b) behavior-based detection from longitudinal transaction trajectories; (c) content-based detection using natural language processing (NLP) of HTML, social media, and SMS text; (d) graph-based detection modeling user-interface transitions and server metadata with heterogeneous graph neural networks; (e) multimodal detection fusing visual features from screenshots with text extracted by optical character recognition (OCR).

Figure 1c depicts the transition to content-based systems, where specialized natural language processing (NLP) models, such as IndoBERT, analyze semantic patterns in HTML, social media, or SMS spam. These models detect local slang such as “ *gacor*,” but they are vulnerable to text obfuscation and keywords hidden in images. To address these structural evasions, Figure 1d illustrates a graph-based approach that maps relationships between UI transitions and server metadata using an HGNN. By modeling the “fake-normal” structure of gambling apps, this method identifies illicit services through structural signatures rather than visible content alone.

Finally, Figure 1e shows the Multimodal architecture that serves as the most comprehensive defense. Cross-modal fusion resolves inconsistencies by integrating visual features from screenshots with semantic features extracted via OCR. For instance, if promotional text is embedded in an image to avoid text-based filters, the visual modality triggers a detection alert. This cross-checking between modalities explains why multimodal systems reported the highest detection scores against content obfuscation among the included studies. However, their resilience to operators who adapt to the detector has not been tested systematically.

Although current detection systems have achieved high accuracy, substantial gaps remain in developing truly robust systems against dynamic evasion tactics. One major obstacle is data drift and temporal instability, where initially accurate models lose sensitivity over time as betting patterns change drastically and slang such as “ *gacor*” evolves for promotions. ^47^ ^,^ ^63^ Furthermore, perpetrators have developed highly sophisticated obfuscation techniques, such as hiding keywords in images, manipulating URL structures to make them unreadable by machines, and using “fake-normal” sites that display clean content but hide gambling payloads. ^48^ ^,^ ^49^ ^,^ ^52^ Another gap is infrastructure reliability: detection often fails because gambling domains have a very short lifecycle, averaging only 24 days before switching to a new backend to avoid being blocked. ^51^ ^,^ ^61^ Finally, most state-of-the-art models remain black boxes, creating interpretability challenges for law enforcement and limiting their ability to provide human-understandable justifications for each detection decision. ^64^

Online gambling threats have evolved from static websites to complex, adaptive ecosystems. Recent studies in 2025 indicate that the primary challenge is no longer simply content classification but the forensics of illicit fund flows and identifying actors’ roles in decentralized networks. However, real-world application is hampered by data scarcity and dynamic slang, necessitating semi-supervised learning approaches, such as that applied to IndoBERT by Santika et al. ^54^

### Related work

The technological landscape of Online Gambling Detection Systems (OGDS) has shifted from passive content blocking to active forensics and behavioral infrastructure analysis. This evolution reflects a shift from rudimentary keyword filtering to sophisticated, multi-layered frameworks that integrate network topology, deep traffic inspection, and psychological markers.

Recent advancements have shifted focus from merely blocking sites to understanding the underlying infrastructure and user behaviors associated with illegal gambling. In web forensics, Chen et al. ^65^ proposed a crawler-based digital forensic method focused on illegal websites. Unlike traditional detection, which stops at the interface level, their approach extracts peripheral information, such as IP addresses, network topology, and server locations, to reconstruct the hidden infrastructure that supports these illicit platforms. Likumahua et al. ^66^ applied live, memory, and network forensics to Indonesian online gambling cases and reported encryption and dynamic IP addresses as key obstacles for investigators. Chen et al. ^67^ crawled 23,142 online gambling websites, clustered them with DBSCAN, and automated the collection of bank account information to trace illicit money flows. Meanwhile, Wang and Shang ^68^ proposed a rapid verification method for black- and gray-industry domain names based on domain name pools, graph computing, image similarity, and clustering. Li et al. ^69^ modeled candidate pornographic and gambling pages and their resources as a heterogeneous information network and reported 83.5% accuracy on 10,033 pages, including pages missed by commercial products, and Zhang et al. ^70^ reported 99.3% accuracy with a fusion-assisted multimodal large language model that combines webpage text, visual content, and embedded image text. Visits to gambling websites are also among the behavioral factors associated with higher odds of malware encounters in enterprise telemetry. ^71^

Complementing these forensic approaches, research has focused on operational efficiency. Erawan et al. ^3^ introduced lightweight scripts that consumed less than 20MB of memory to detect hidden page injections on government servers. Harahap and Ridho ^2^ used attack signatures to detect defacement. To accelerate reporting, systematic URL extraction from search-engine results reached 89% accuracy, compared with 53% for manual search without content verification, ^60^ and an automated registration framework acquired 7,496 fund accounts and identified 23 criminal gangs operating behind hundreds of domains. ^6^ Zhang et al. ^72^ focused on identifying gambling websites by analyzing user behavior and site templates. Utilizing an improved Agglomerative Clustering algorithm combined with heap optimization, they analyzed cookie parameters and dataset features to categorize illegal gambling sites into specific types, such as board games, virtual lotteries, and sports betting. Feng et al. ^73^ identified anomalous POST traffic based on gambling website templates, Kurniawan and Triayudi ^74^ combined Wazuh and Velociraptor to reconstruct gambling web defacement attacks, and Ghani et al. ^75^ combined Isolation Forest and Random Forest to detect DDoS, malware, and backdoor attacks.

The transition to graph-based detection has become essential for countering sophisticated evasion tactics in mobile environments. Ma et al. ^52^ demonstrated that integrating content features with structural information via HGNN is highly effective in identifying websites masquerading as legitimate portals. Lightweight graph-based methods for imperfect datasets, ^76^ multi-level feature fusion, ^77^ and dual-learning ensembles ^78^ have likewise been applied to gambling and pornographic website classification. In the mobile app domain, Lu et al. ^53^ (TacDroid) and Chen et al. ^58^ (DeUEDroid) utilize User Interface (UI) transition analysis to detect illegal functionalities hidden within dynamic WebViews. Gu et al. ^51^ (HeCGamb) utilized encrypted traffic analysis to map communication patterns between mobile applications and the broader gambling industry chain, thereby providing a holistic view of the illicit ecosystem. Earlier measurement studies established the scale of this ecosystem: Gao et al. ^79^ collected 3,366 illegal gambling apps in China, characterized their covert distribution channels and abused fourth-party payment services, and identified more than 140,000 suspicious gambling domains through guilt-by-association expansion; Han et al. ^80^ built a knowledge graph from 277,816 domains to show how dark links and black-hat search engine optimization promote gambling apps; and Hong et al. ^10^ analyzed ground-truth data on 1,461 scam incidents and 1,487 gambling scam apps, revealing the abuse of public app generators and money-mule payment channels. Blancaflor et al. ^81^ further examined the security risks of Android gambling apps downloaded from unofficial sources. On social media, Costa et al. ^82^ framed YouTube comments on the Fortune Tiger game in Brazil as a stance-detection task and found that favorable comments were frequent, repetitive, and possibly automated.

Behavioral and psychometric indicators provide complementary evidence on gambling-related harm, although they concern users rather than services. Ghaharian et al. ^45^ and Stechschulte et al. ^56^ established that transaction logs, such as deposit frequency and total losses, are critical indicators for identifying high-risk gambling subgroups. Complementing these technical methods, Ling et al. ^83^ applied network analysis to survey data from 699 adult loot-box purchasers and reported a significant positive association between gambling disorder symptom severity and risky loot-box engagement. This suggests that risk-screening components of OGDS could consider behavioral risk markers in convergence areas, such as online gaming, to identify early signs of gambling-related harm. ^44^ ^,^ ^46^ Related work has linked problem-gambling message-board activity to the legalization of sports betting in the United States, ^84^ examined whether user activity promotes gambling-related content on Twitter/X, ^85^ applied hybrid neural networks to customer churn prediction in web services, ^86^ and analyzed digital risks and self-regulation in the international spread of gambling addiction. ^87^

In decentralized ecosystems, Wang et al. ^88^ combined community detection and node2vec embeddings to identify gambling addresses and transactions, with LightGBM reaching an accuracy of 0.84; Duan et al. ^89^ proposed VetSC to vet the safety of smart contracts in decentralized applications and uncovered risks such as expired lottery tickets; and Carvalho ^90^ coded loot boxes as smart contracts to address transparency and trust problems in gambling-like game mechanics. Huang et al. ^91^ used multimodal retrieval to find cryptocurrency gamblers, Wu et al. ^92^ applied community detection to blockchain social networks, and Datta et al. ^93^ designed a secure and fair online lottery system based on smart contracts.

Although recent studies have introduced sophisticated domain-specific models, such as heterogeneous graphs for mobile apps ^51^ and websites ^52^ and role identification in decentralized crypto-gambling, ^94^ these approaches remain fragmented across platforms. Musa et al. ^95^ assessed cyber vulnerabilities of a legal operator, the Croatian Lottery, through open-source intelligence rather than detecting illegal gambling services; Hu and Yuan ^96^ reviewed data-driven approaches to malicious website detection in general; Das et al. ^97^ surveyed attacks on blockchain systems; and Šarčević et al. ^98^ reviewed predictive modeling of tennis matches. Reviews from the behavioral sciences examined the relationship between videogame micro-transactions and problem gambling, ^99^ internet addictions outside Europe, ^100^ problematic internet use among university students, ^101^ the association between adult attachment and problematic internet use, ^102^ and publication trends in internet addiction research. ^103^ Closer to the harm domain of this review, two scoping reviews synthesized data-science and risk-assessment models that operators use to identify players at risk, ^104^ ^,^ ^105^ and a survey published after our search date covered AI-based detection of illicit web ecosystems, including illegal gambling websites, alongside phishing and scam sites. ^106^ Table 2 compares these secondary studies with the present review: each addresses a single modality, operator security, betting prediction, or the behavioral correlates and player-level risk of problematic gambling and internet use, and none synthesizes the detection of illegal online gambling services across platforms. To our knowledge, this is the first PRISMA-guided systematic review restricted to online gambling detection systems that jointly covers website and mobile detection, social media promotion, ^4^ ^,^ ^54^ infrastructure-level evasion such as domain name system (DNS) clustering, ^1^ harm detection, and blockchain forensics, and that appraises how the reported performance was obtained. Furthermore, researchers often neglect the temporal sustainability of these models; shifts in gambler behavior, or “data drift,” can degrade performance if decision thresholds are not recalibrated periodically. ^63^ This absence matters because detection evidence is scattered across web security, mobile software engineering, natural language processing, and blockchain analytics venues, so a regulator or system designer cannot judge which architecture suits which platform, or how far the reported scores can be trusted. This review addresses that gap in two ways: it integrates the fragmented modalities into a single cross-platform taxonomy and research roadmap, and it appraises how the reported performance was obtained, which determines whether those scores can inform deployment.

### Objectives and contributions

To address these critical gaps, this study presents a Systematic Literature Review (SLR) of 39 primary studies published between 2021 and 2025. The contributions of this study are fivefold.

First, we propose a cross-platform taxonomy that classifies 39 detection studies into six operational domains (website infrastructure, mobile ecosystems, social media platforms, gambling-related harm and risk profiling, decentralized blockchain networks, and network infrastructure) and six architectural integration patterns. Existing reviews treat malicious or illicit websites, blockchain attacks, or player-level gambling risk separately ( Table 2); this taxonomy frames them together and links each input modality to the task it serves and the operational use of its output.

**Figure 2.**
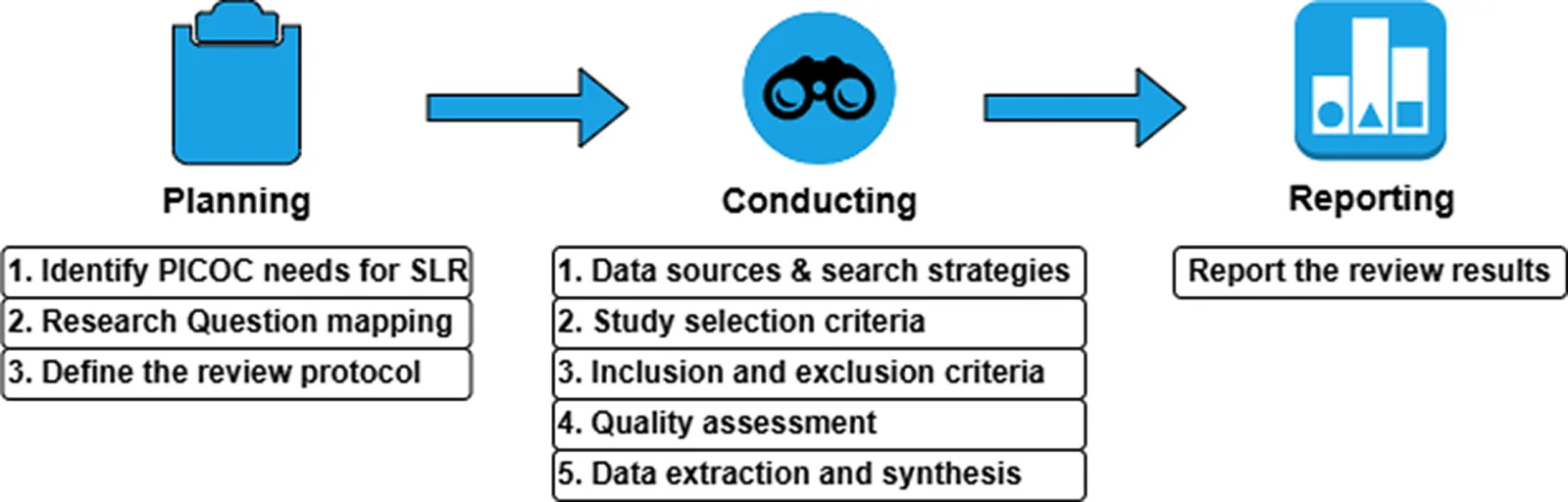
Three-phase systematic literature review process based on the PICOC framework. Planning (left): identification of PICOC needs, research question mapping, and protocol definition. Conducting (center): data sources and search strategy, selection criteria, inclusion and exclusion criteria, quality assessment, and data extraction and synthesis. Reporting (right): reporting of the review results. PICOC: population, intervention, comparison, outcome, and context; SLR: systematic literature review.

**Figure 3.**
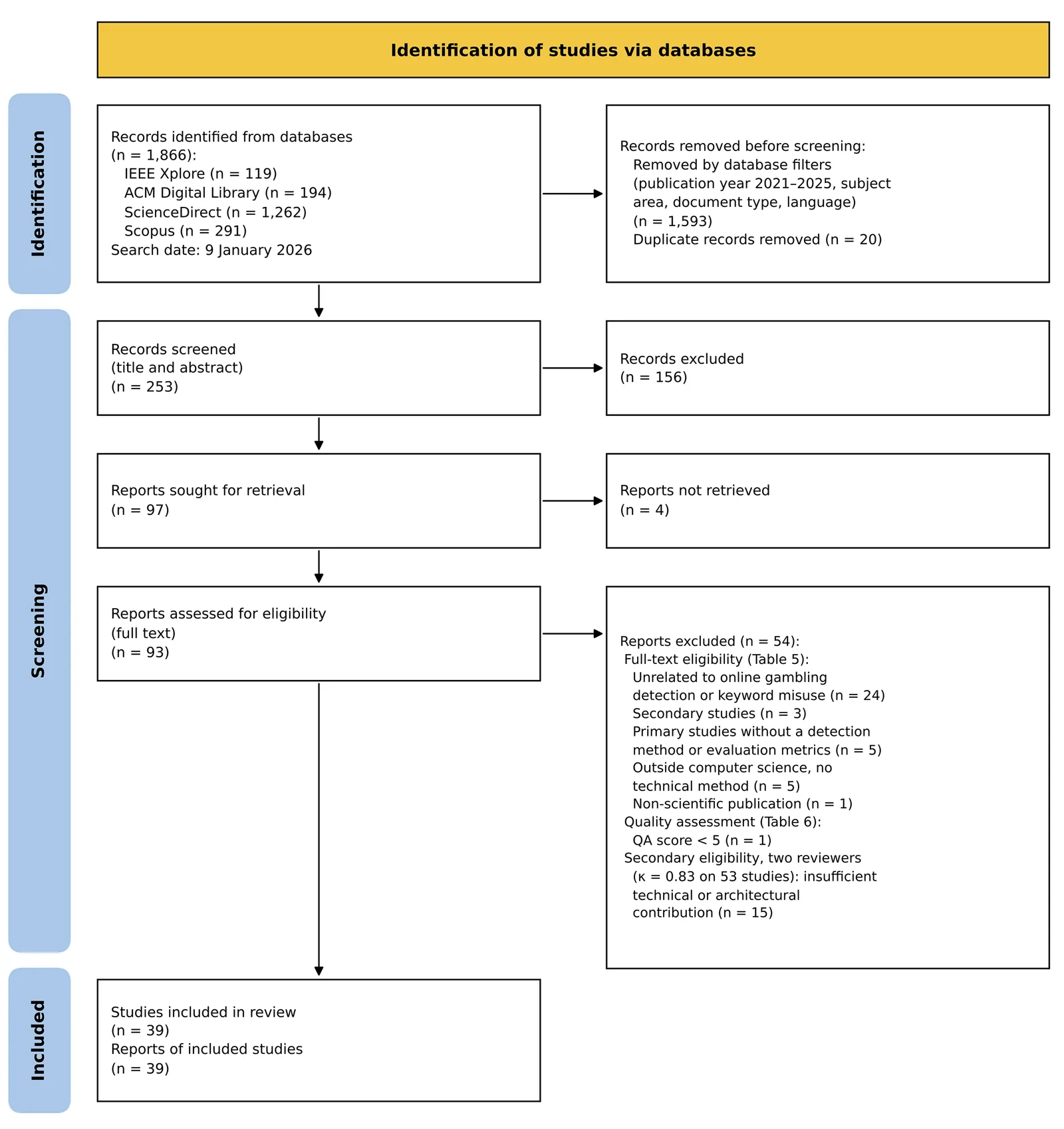
PRISMA flow diagram of study identification, screening, and inclusion. We identified records in IEEE Xplore, the ACM Digital Library, ScienceDirect, and Scopus. Counts correspond to the screening, quality assessment, and final inclusion worksheets of the underlying data. PRISMA, Preferred Reporting Items for Systematic Reviews and Meta-Analyses; QA, quality assessment.

**Figure 4.**
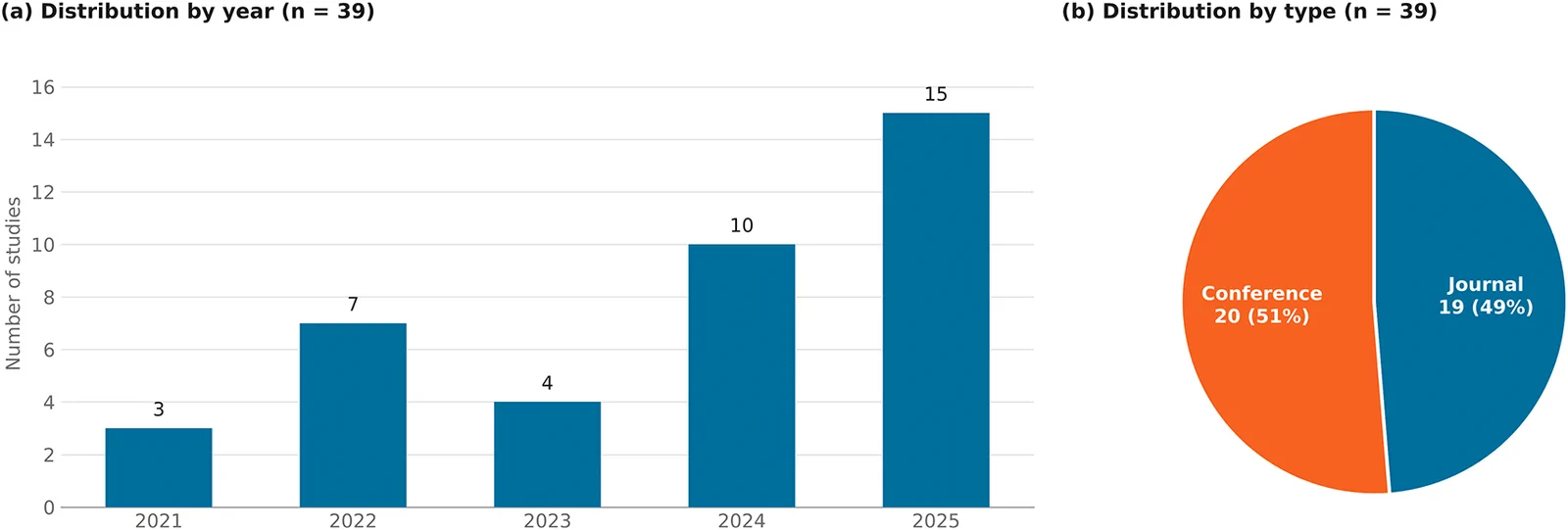
Publication year and publication type of the 39 primary studies. (a) Number of included studies by publication year (2021–2025); (b) proportion of conference papers and journal articles.

**Figure 5.**
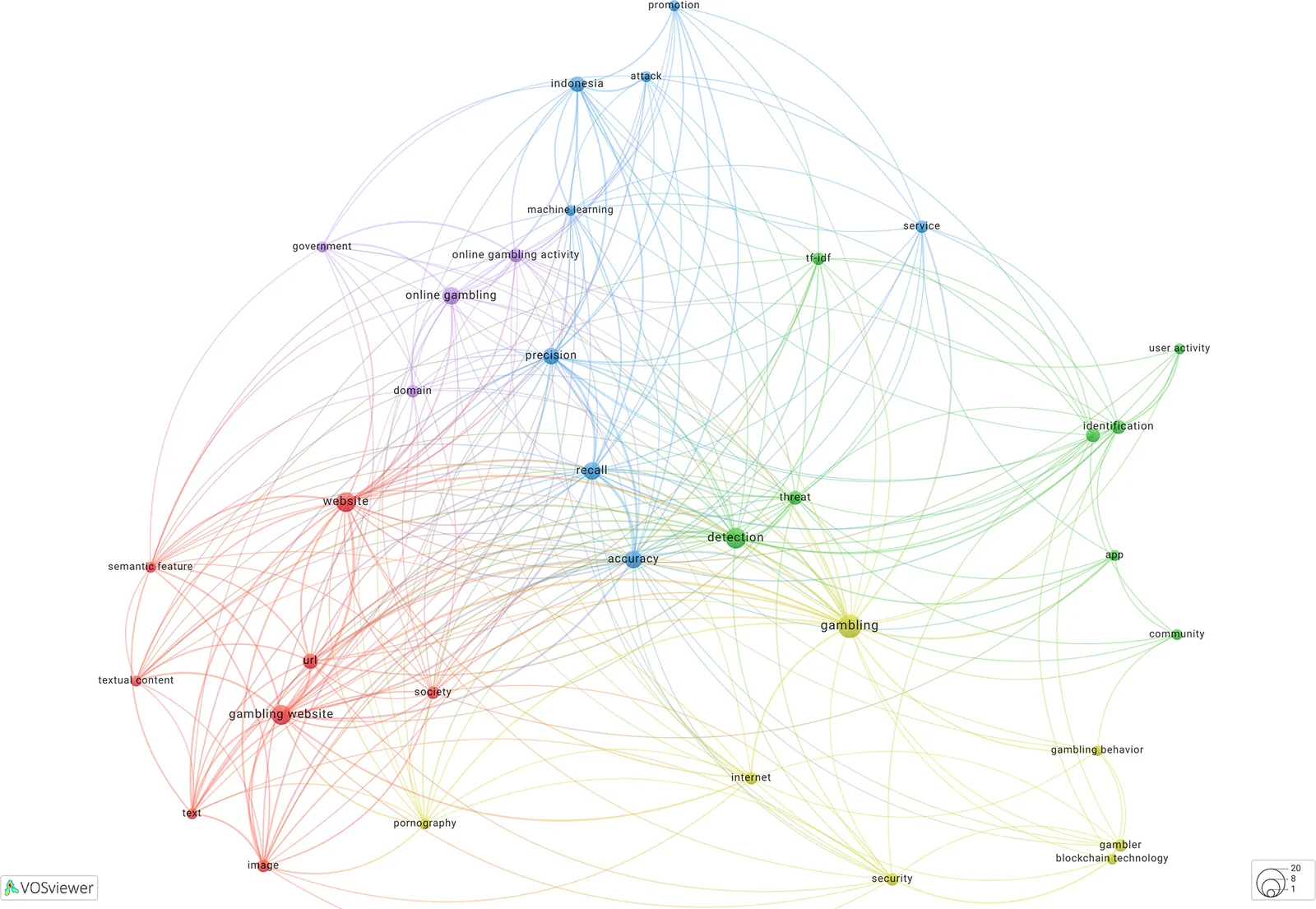
Term co-occurrence map of the 39 included studies created in VOSviewer Online 1.2.4. Terms were extracted from titles, abstracts, and author keywords after stop-word removal and lemmatization; node size is proportional to term frequency, and links indicate co-occurrence. [VOSviewer 1.2.4.]

**Figure 6.**
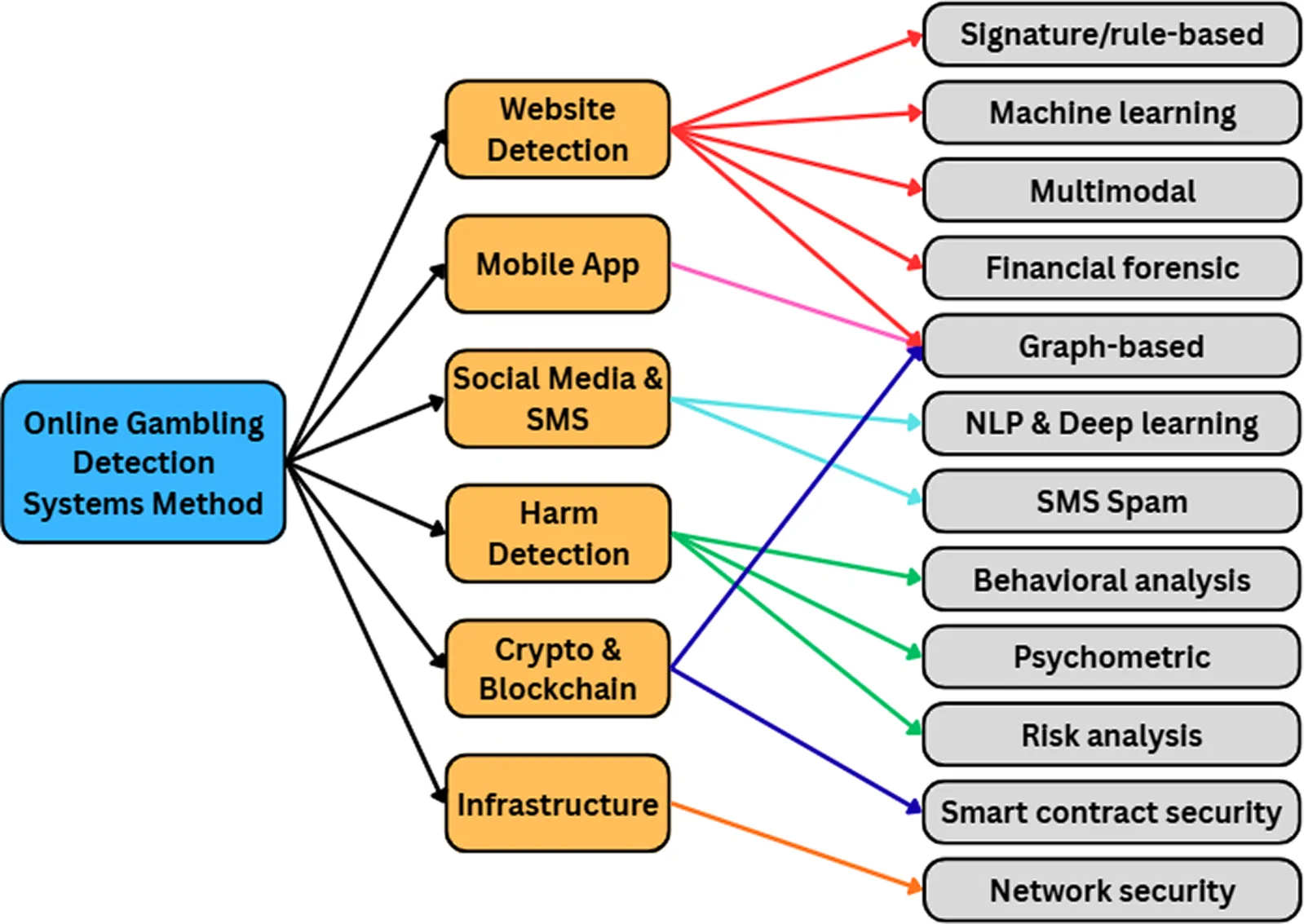
Mapping of detection domains to task and method categories. Six application domains (website detection, mobile app, social media and SMS, harm detection, crypto and blockchain, infrastructure) are linked to the method categories used in the primary studies. NLP, natural language processing; OGDS, online gambling detection systems; SMS, short message service.

**Figure 7.**
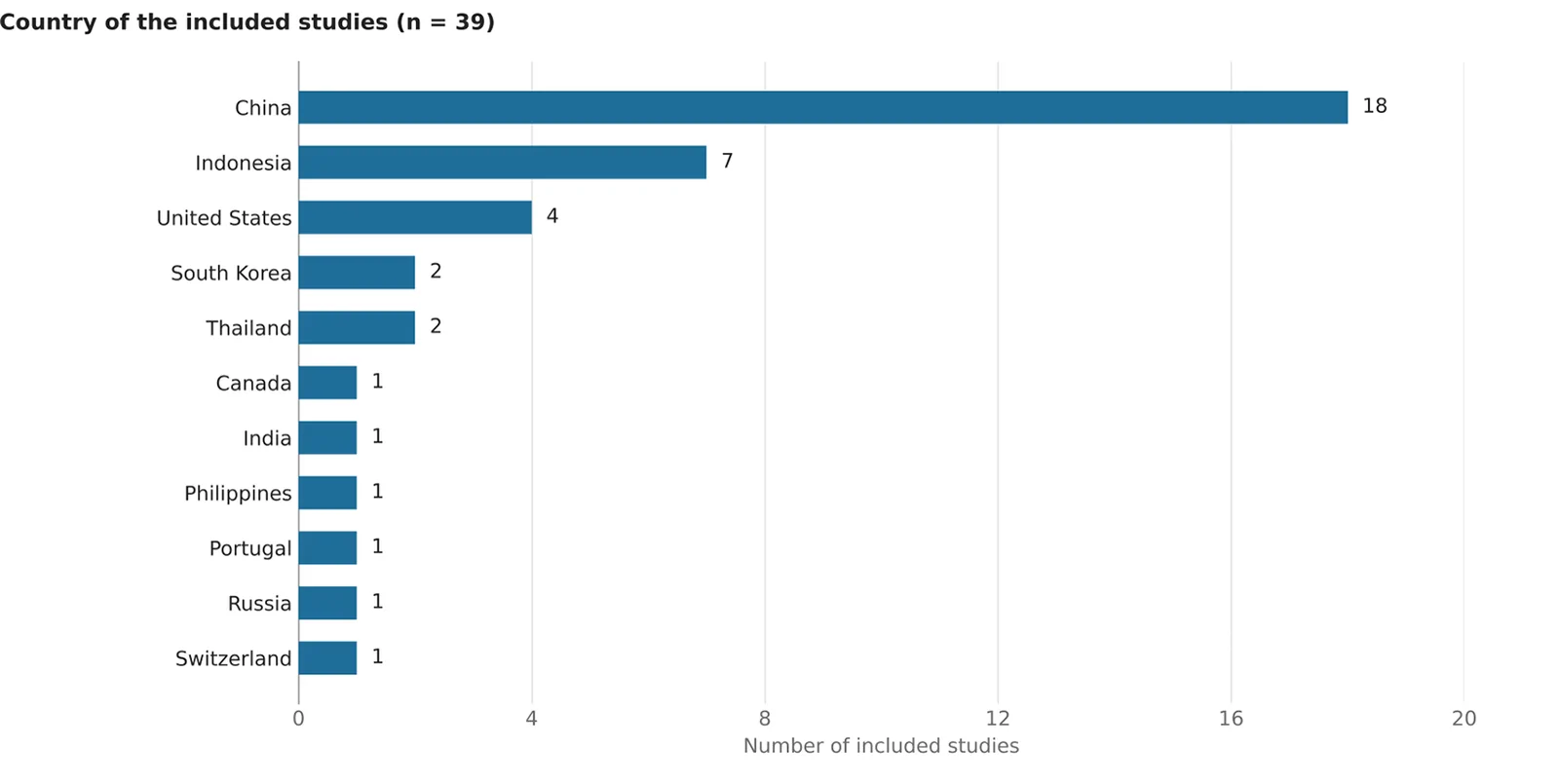
Geographic distribution of the 39 primary studies by country. Counts reflect the geographical scope or origin of each study as extracted in Table 10.

**Figure 8.**
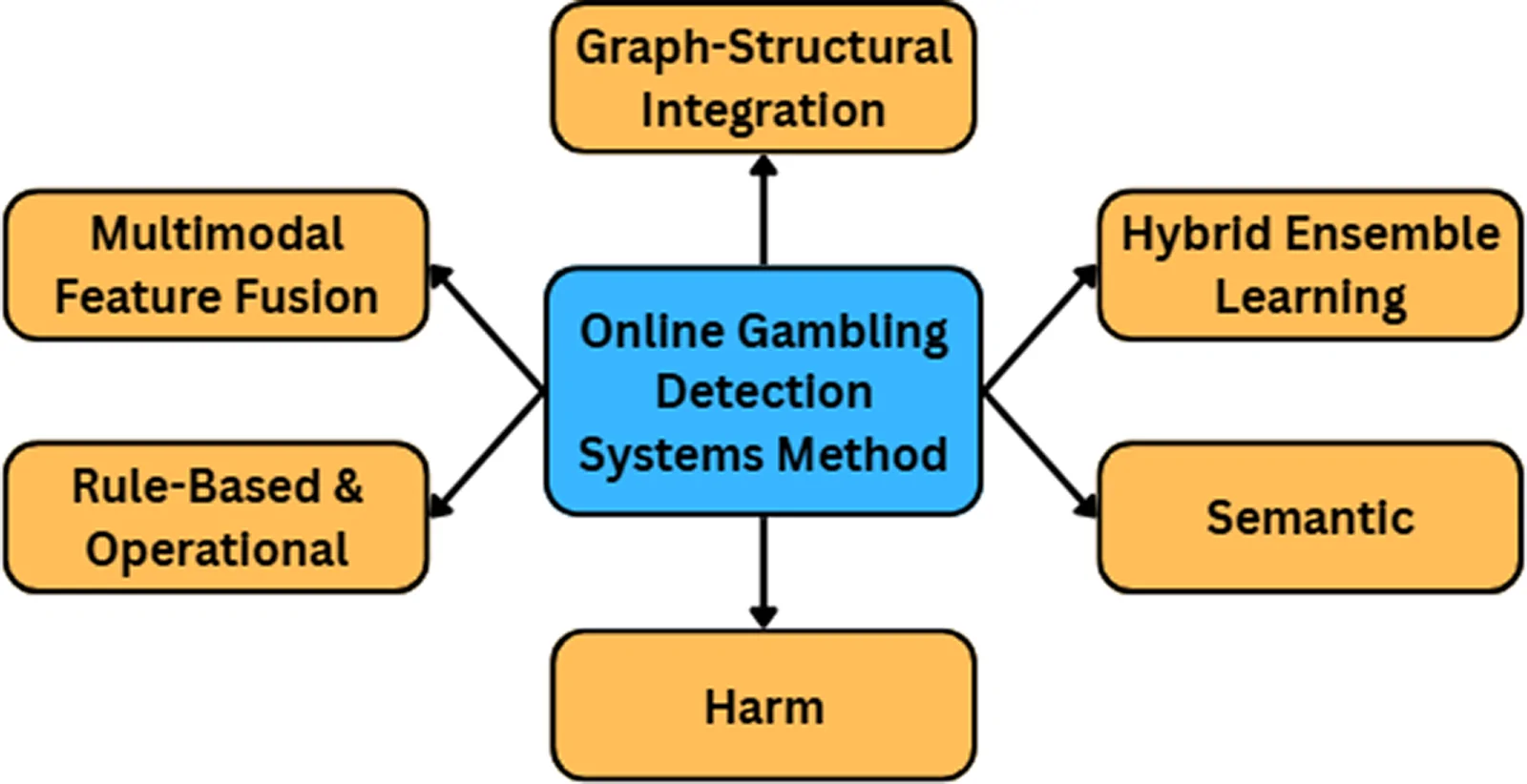
Six architectural integration patterns of online gambling detection systems. Multimodal feature fusion, graph-structural integration, hybrid ensemble learning, semantic, harm, and rule-based, and operational systems (see Table 12).

**Figure 9.**
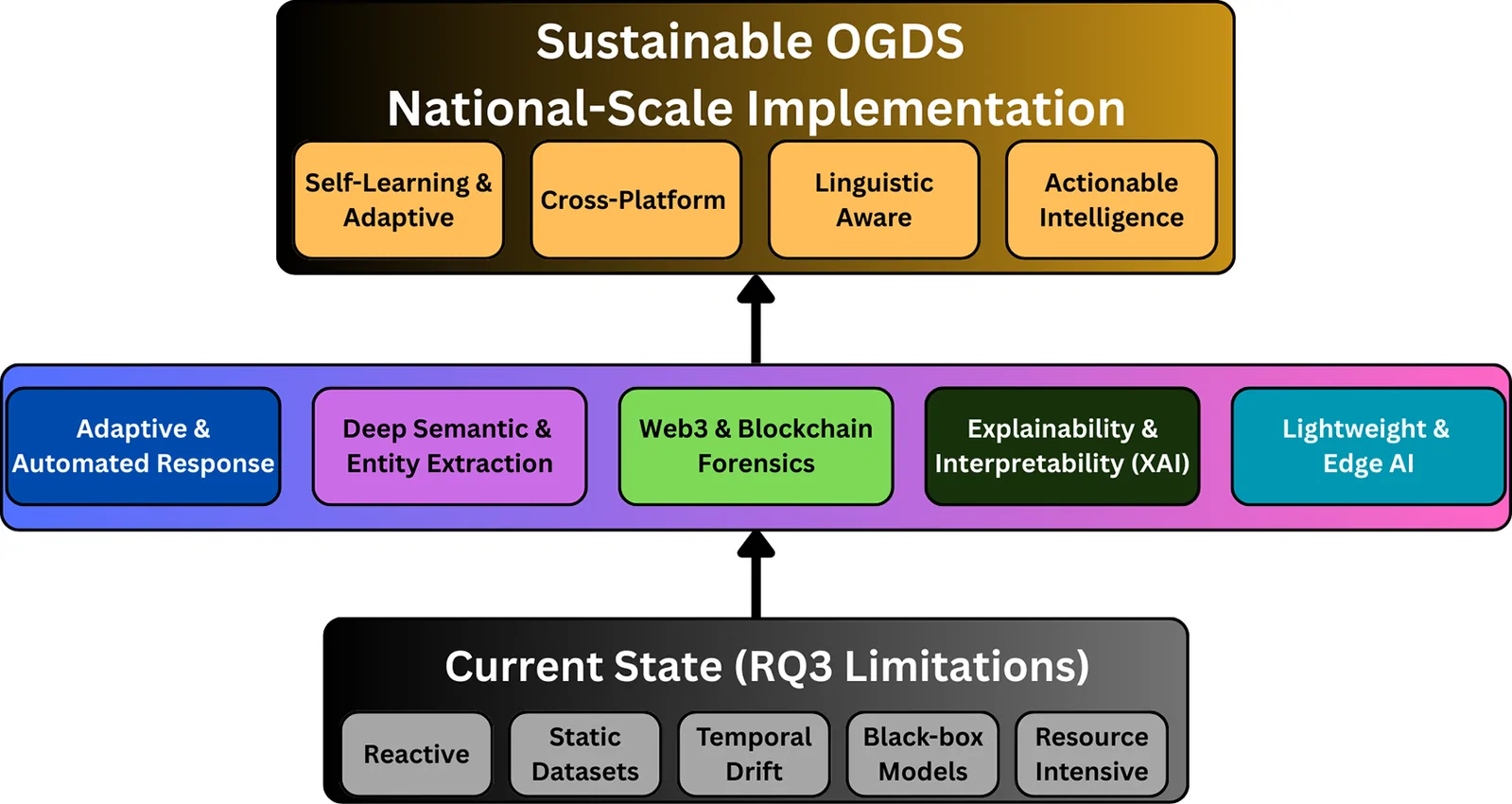
Roadmap of emerging trends and future directions in online gambling detection. Current limitations identified in RQ3 (bottom) are linked through five research pillars (middle) to sustainable national-scale implementation (top). OGDS, online gambling detection systems; RQ, research question; XAI, explainable artificial intelligence.

**Figure 10.**
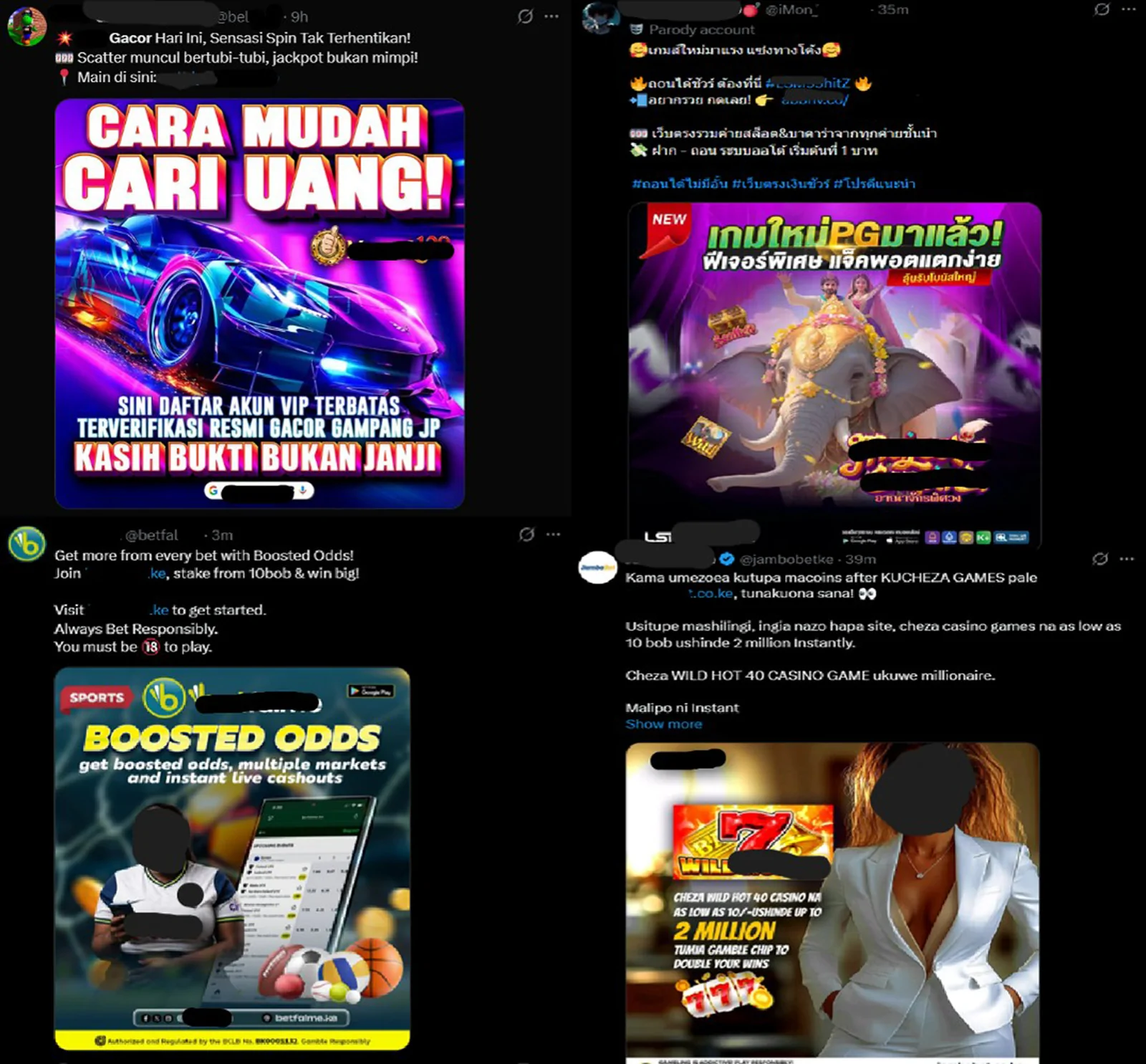
Examples of watermarked advertising images used to promote online gambling. Screenshots of social media posts in which promotional text is embedded in images, separating visual signals from textual cues. All account names, usernames/handles, profile identifiers, URLs, and other potentially identifying information have been fully obscured to prevent identification.

**Figure 11.**
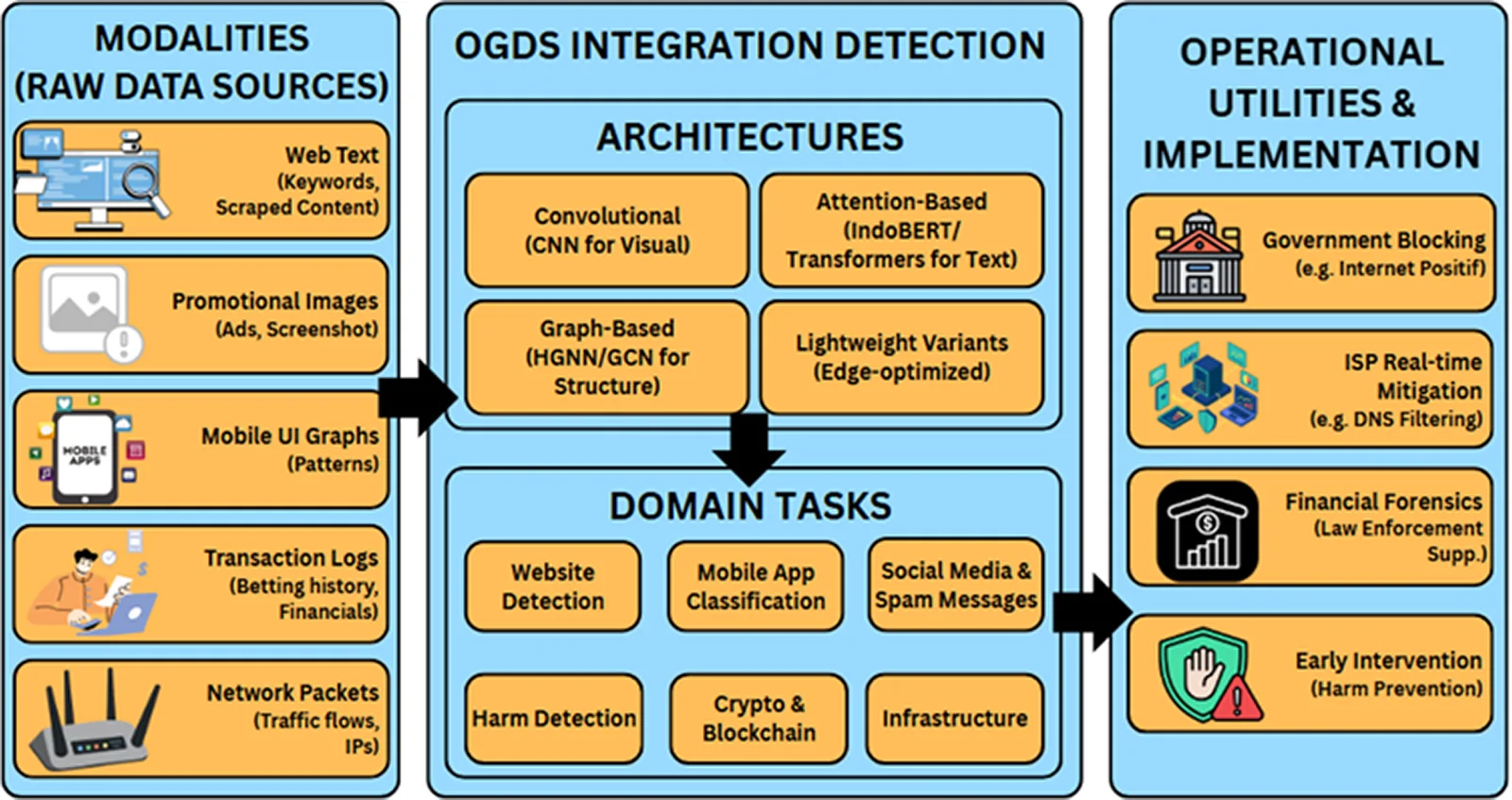
Unified workflow of AI-based online gambling detection systems. Detection architectures (convolutional, attention-based, graph-based, lightweight variants) process input modalities (web text, promotional images, mobile UI graphs, transaction logs, network packets) for domain tasks, and their outputs support operational utilities (government blocking, ISP real-time mitigation, financial forensics, early harm intervention). CNN, convolutional neural network; GCN, graph convolutional network; HGNN, heterogeneous graph neural network; ISP, internet service provider; UI, user interface.

Second, we provide a structured comparison of reported performance across domains and assess the trade-off between detection accuracy and computational cost. Because the primary studies used different datasets, splits, and metrics, this comparison is descriptive rather than a meta-analysis.

**Table 2.** Comparison of this review with related secondary studies.

Study	Design and scope	Domains	Primary focus	Gap relative to this review	Excluded at
**Musa et al., 2024** ^95^	Survey (book chapter) with an OSINT case study; cybersecurity risks of a legal online lottery operator	–	Operator-side vulnerability assessment	Operator security rather than detection of illegal services	Full text
**Hu and Yuan, 2023** ^96^	Narrative review; data-driven malicious-website detection	W (general)	URL, content, and learning-based features	Gambling not treated as a distinct target; no M, S, N, H, or B	Title and abstract
**Das et al., 2025** ^97^	Survey; layer-based taxonomy of attacks on blockchain systems	B (security)	Attack vectors and defenses	No gambling-related detection	Title and abstract
**Šarčević et al., 2022** ^98^	Review; predictive modeling of tennis matches	–	Outcome prediction relevant to betting	Prediction rather than detection	Title and abstract
**Gibson et al., 2022** ^99^	Systematic review; 19 studies from four databases	H	Videogame micro-transactions and problem gaming and gambling	Behavioral correlates; no detection method or performance metrics	Full text
**Kuss et al., 2021** ^100^	Systematic literature review; 64 studies from outside Europe	–	Prevalence and correlates of internet addictions	No detection of gambling services or harm	Full text
**Sánchez-Fernández et al., 2023** ^101^	Systematic review; problematic internet use in university students	–	Predictive factors	No detection of gambling services or harm	Title and abstract
**Niu et al., 2023** ^102^	Three-level meta-analysis; adult attachment and problematic internet use	–	Pooled association	No detection of gambling services or harm	Title and abstract
**Muflih et al., 2024** ^103^	Bibliometric analysis; internet-addiction publications, 1996–2022	–	Publication trends	Bibliometric mapping only	Title and abstract
**Ghaharian et al., 2023** ^104^	Scoping review (PRISMA); 37 studies	H	Data science for responsible gambling (clustering, supervised learning)	Player-level risk only; no service detection	Not retrieved
**Marionneau et al., 2025** ^105^	Scoping review (PRISMA-ScR); 31 academic models and 11 commercial tools	H	Operators’ risk-assessment models under duty of care	Player-level risk only; no service detection	Not retrieved
**Hwang and Min, 2026** ^106^	Structured survey without a registered protocol; AI-based detection of illicit web ecosystems (phishing, illegal gambling, scams, malvertising)	W, N	Text, vision, graph, and transformer detectors; datasets	Gambling is one of several threats; mobile apps, messaging, blockchain, and harm not synthesized	Published after search date
**This review**	Systematic review (PRISMA 2020); 39 studies, 2021–2025, four databases	W, M, S, N, H, B	Detection of illegal online gambling services and gambling-related harm	–	–

*Note*: W, website; M, mobile application; S, social media and messaging; N, network infrastructure; H, gambling-related harm and risk profiling; B, blockchain and cryptocurrency; −, none; OSINT, open-source intelligence; PRISMA-ScR, PRISMA extension for scoping reviews. “Excluded at” gives the screening stage at which a study was excluded from this review; the three studies excluded at full text were classified as secondary studies. “General” and “security” indicate coverage that is not specific to gambling. The three studies identified during revision were not retrieved by our search; Hwang and Min was published after the search date (9 January 2026).

**Table 3.** PICOC framework defining the scope of the review.

Component	Description
Population (P)	Online gambling systems related to illegal online gambling activities
Intervention (I)	Implementation of detection systems
Comparison (C)	Have a baseline method or reference approaches
Outcome (O)	Improved detection accuracy and robustness to evasion
Context (C)	Research within the field of computer science

**Table 4.** Research questions and objectives.

No.	Research question	Objective
RQ1	How has the system been used and utilized in various modalities and tasks of online gambling detection?	Cataloging and taxonomy of various studies that integrate the online gambling detection pipeline, covering a wide range of tasks such as defacement detection, social media promotion, and addiction analysis in various modalities (text, visual, behavioral, and network traffic)
RQ2	How can existing systems be integrated with other architectures to improve performance in online gambling detection tasks?	Presents a quantitative synthesis of performance trends across architectures, such as the use of hybrid models that combine automated crawling with advanced deep learning to surpass the efficiency of traditional methods
RQ3	What are the main challenges and limitations associated with implementing an online gambling detection system?	Identify key barriers to building resilient systems, including data drift phenomena, temporal instability, obfuscation techniques, very short domain life cycles, and interpretability challenges in models
RQ4	What are the emerging trends and future directions for online gambling detection systems?	Formulate open challenges and future research directions so that researchers can design detection systems that are not only statistically accurate, but also resistant to data manipulation and dynamic changes in perpetrator behavior

**Table 5.** Inclusion and exclusion criteria.

Component	Inclusion criteria	Exclusion criteria
Publication type	Journal and conference papers indexed in reputable sources	News articles, blogs, opinion papers, theses, or non-scientific publications
Publication date	Published within 2021–2025	Published before 2021
Relevance to topic	Keywords related to online gambling and detection appear in title, abstract, or author keywords	Topic unrelated to online gambling detection systems or misuse of the keywords
Evaluation metrics	Presents measurable evaluation metrics	Does not have evaluation metrics
Subject area	Computer science or closely related fields	Domains outside computer science without a technical method
Study design	Primary studies proposing or evaluating a method or system	Secondary studies (surveys or reviews)
Accessibility	Full text is accessible	Full text is not accessible

**Table 6.** Quality assessment checklist.

No.	Assessment question	Answer	Score
Q1	Does the study apply any technical techniques?	Yes/No	1/0
Q2	Does the study provide details about the dataset?	Yes/No	1/0
Q3	Does the study describe the preprocessing or feature extraction methods?	Yes/No	1/0
Q4	Does the study describe the system architecture details?	Yes/No	1/0
Q5	Does the study present evaluation performance metrics?	Yes/No	1/0
Q6	Does the study mention bias control, data validation, scalability, or reliability measures?	Yes/No	1/0
Q7	Does the study include comparison with baseline methods?	Yes/No	1/0
Q8	Are the study results better than the baseline?	Yes/No/N/A	1/0
Q9	Does the study discuss limitations, challenges, or evasion strategies?	Yes/No	1/0
Q10	Does the study suggest directions for future work or improvements?	Yes/No	1/0

**Table 7.** Quality score distribution of the 54 studies that passed quality assessment.

Quality category	Score range	Count	Percentage
High	9–10	22	40.7%
Moderate	6–8	26	48.1%
Low	5	6	11.1%
**Total**		**54**	**100%**

**Table 8.** Agreement matrix between the two reviewers for the 53 assessed studies.

Reviewer A/Reviewer B	Include	Exclude	Total
Include	34	0	34
Exclude	4	15	19
**Total**	38	15	53

**Table 9.** Observed and expected agreement used to compute Cohen’s kappa.

Component	Value
Observed agreement ( *P*_o_)	0.925
Expected agreement ( *P*_e_)	0.561
**Cohen’s kappa (κ)**	**0.83**

**Table 10.** Data extraction fields and their mapping to the research questions.

Extraction field	Description	RQ
Application field/domain	Target application domain of the detection system	RQ1
Algorithm or method used	Core detection approach employed in the study	RQ1, RQ2
Dataset and modality	Dataset characteristics and modality used for detection, such as textual content, images, videos, user behavior logs, network traffic, or multimodal data	RQ1
Scope/author region	Geographical scope or origin of the study, including regulatory or cultural context influencing system design	RQ1
Gaps and challenges	Identified technical and operational limitations, such as data imbalance, concept drift, obfuscation techniques, short domain lifecycles, or lack of interpretability	RQ3
Findings in research	Key empirical findings and performance outcomes reported by the study, highlighting effectiveness, scalability, or comparative advantages over baseline approaches	RQ2
Evaluation/performance metrics method	Evaluation protocols and metrics applied	RQ2
Future directions and trends	Emerging research directions proposed by the authors	RQ4

**Table 11.** Research distribution of the 39 primary studies by domain, problem focus, and method.

Domain	Problem focus	Approach	Task and method
**Website detection**	Detection of gambling websites, web defacement on academic and government domains (.ac.id/.go.id), and “fake-normal” sites	*Signature/rule-based *	Suricata IDS, ^2^ Bash script, ^3^ BeautifulSoup, ^60^ Wazuh forensics ^74^
		*Machine learning*	Hybrid ensemble, ^78^ Co-training, ^49^ SVM (PSO-optimized), ^73^ Identification models ^72^
		*Multimodal*	MEDAL, ^62^ DRSDetector, ^77^ HGNN, ^52^ Lightweight graph, ^76^ Vision–language model, ^59^ Hybrid fusion ^50^
		*Financial forensic*	Illegal fund and account analysis ^6^
**Mobile app**	Illegal Android/iOS gambling applications, underground distribution, repackaging, and promotion via messaging platforms	*Graph-based detection*	TacDroid (UTG), ^53^ DeUEDroid, ^58^ HeCGamb (heterogeneous graph) ^51^
**Social media and spam messages**	Gambling promotion on YouTube, Twitter, and SMS-based spam campaigns	*NLP and deep learning*	IndoBERT, ^47^ ^,^ ^54^ Large-scale Twitter analysis ^4^ ^,^ ^85^
		*SMS spam detection*	Honeypot with RTT, ^48^ SMS-based IOG detection ^57^
**Harm detection**	Detection of gambling addiction, behavioral profiling, and continuous risk prediction	*Behavioral analysis*	In-database feature extraction, ^56^ Temporal model stability, ^63^ Transaction clustering ^45^
		*Psychometric*	UTAUT3, ^46^ Psychometric network analysis, ^83^ Interrupted time series (Reddit) ^84^
		*Risk analysis*	Churn prediction models, ^86^ Time-series analysis, ^44^ Explainable XGBoost with LLM-based RAG ^64^
**Crypto and blockchain**	Cryptocurrency-based gambling platforms and smart contract ecosystems	*Graph mining*	Multimodal retrieval, ^91^ Role identification, ^94^ Community detection ^92^
		*Smart contract security*	Secure lottery design ^93^
**Infrastructure**	Network-level mitigation and financial flow analysis of illegal gambling	*Network security*	DNS RPZ clustering, ^1^ AI-driven network security ^75^

**Table 12.** System-level architectural integration patterns in the 39 primary studies.

Category	Typical design pattern	No. of papers
Multimodal feature fusion systems	Integration of visual (ResNet, VGG) and textual (Bi-LSTM, OCR) features using co-training, tri-training, or transformer-based attention to bridge the semantic gap	7
Graph-structural integration systems	Construction of UTG or HGNN to model navigation paths, traffic flows, and transaction networks	7
Hybrid ensemble learning systems	Combining multiple ML algorithms (e.g., Isolation Forest + Random Forest, CNN + LSTM + GNN) or optimizing parameters using meta-heuristic algorithms such as PSO	4
Semantic systems	Leveraging pre-trained transformers (IndoBERT, multilingual) and NLP techniques to handle linguistic complexity, slang, and obfuscated URLs in social media and SMS	5
Harm systems	Longitudinal data analysis using time-series clustering, transaction ratio monitoring, and psychometric models (UTAUT3) to identify addiction and harm, and explainable risk classification that combines XGBoost with LLM-based RAG	9
Rule-based and operational systems	Integration of detection logic with practical tools such as IDS (Suricata/Wazuh), Telegram bots, file system monitors (inotify), and blockchain smart contracts	7
**Total**		**39**

**Table 13.** Reported performance of methods in the website detection domain.

Method (task)	Acc (%)	Prec (%)	Rec (%)	F1 (%)	Other/specific metrics
Suricata IDS ^2^ (signature/rule-based)	97	–	–	97	TPR: 95%, TNR: 99%, PPV: 99%, NPV: 96%
BeautifulSoup ^60^ (signature/rule-based)	89.22	83.08	100	90.76	TP: 1242, FP: 253
Bash script ^3^ (signature/rule-based)	–	–	–	–	CPU: 1%, memory: 20 MB
Wazuh forensics ^74^ (signature/rule-based)	–	–	–	–	MTTD: 1.2 s/alert
Hybrid ensemble ^78^ (machine learning)	96.83	–	–	–	–
Co-TextRNN ^49^ (machine learning)	–	98.35	98.33	98.33	–
SVM-PSO ^73^ (machine learning)	99.85	99.85	99.85	99.85	–
Improved agglomerative ^72^ (machine learning)	98.21	–	98.94	–	Silhouette: 0.57
MEDAL ^62^ (multimodal)	99.57	99.44	99.25	99.34	–
DRSDetector ^77^ (multimodal)	97.83	96.47	97.81	–	–
HMAN (HGNN) ^52^ (multimodal)	–	97.6	92.9	95.1	–
Lightweight graph ^76^ (multimodal)	–	93.29	90.8	92.01	CPU time: 4 min 3 s
Vision–language ^59^ (multimodal)	95	–	95	95	–
Hybrid fusion ^50^ (multimodal)	99.31	99.32	99.31	99.31	–
Illegal fund and account UI analysis ^6^ (financial forensic)	–	83	84	83.5	mAP: 83.96%

**Table 14.** Reported performance of methods in the mobile application domain.

Method (task)	Acc (%)	Prec (%)	Rec (%)	F1 (%)	Other/specific metrics
TacDroid (UTG) ^53^ (graph-based detection)	–	–	–	96.73	–
DeUEDroid (MaskGAE) ^58^ (graph-based detection)	98.97	–	–	98.22	–
HeCGamb (HGNN) ^51^ (graph-based detection)	–	100.0	98.80	99.40	AUC: 99.4%; FLOPs/training time: 10.25 M/212.56 s; parameters/test time: 20.15 K/29.69 s; open-world F1: 94.1%, AUC: 96.8%

**Table 15.** Reported performance of methods in the social media and spam message domain.

Method (task)	Acc (%)	Prec (%)	Rec (%)	F1 (%)	Other/specific metrics
IndoBERT ^47^ (NLP and deep learning)	98.53	98.88	95.65	97.24	F-beta score: 98.21
Semi-supervised IndoBERT ^54^ (NLP and deep learning)	98.60	99.10	98.00	98.50	–
Large-scale Twitter analysis ^4^ (NLP and deep learning)	–	98.82	98.80	98.80	–
User activity and gambling content on Twitter/X ^85^ (statistical analysis)	–	–	–	–	p-value: 0.001, Tau-U: −0.71
Honeypot with RTT ^48^ (SMS spam detection)	99.77	84.61	98.33	90.96	–
SMS IOG detection ^57^ (SMS spam detection)	97.88	97.81	97.91	97.86	–

**Table 16.** Reported performance of methods in the harm detection domain.

Method (task)	Prec (%)	Rec (%)	AUC (%)	p-value	Other/specific metrics
In-database feature extraction ^56^ (behavioral analysis)	2.40	84.29	92.58	–	MI: 0.00448
Temporal model stability ^63^ (behavioral analysis)	–	–	–	<0.001	t = 7.21 (ΔAUPRC = +7.06%, PGSI 8+)
Transaction clustering ^45^ (behavioral analysis)	–	–	–	–	Composite index (CI): 1.42
Extended UTAUT3 ^46^ (psychometric)	–	–	–	–	SRMR: 0.066, NFI: 0.913, chi-square: 2.261
Psychometric network analysis ^83^ (psychometric)	–	–	–	–	KR-20: 0.82
Interrupted time series (Reddit) ^84^ (psychometric)	–	–	–	0.001	*R* ^2^: 0.861, estimate: 0.791, SE: 0.073
Churn prediction models ^86^ (risk analysis)	–	–	94.00	–	F1: 67.00
Time-series analysis ^44^ (risk analysis)	–	–	–	–	Silhouette score: 0.693
Explainable XGBoost with LLM-based RAG ^64^ (risk analysis)	–	–	87.00	–	SHAP feature attribution; LLM-generated justifications

**Table 17.** Reported performance of methods in the crypto and blockchain domain.

Method (task)	Acc (%)	Prec (%)	Rec (%)	F1 (%)	Other/specific metrics
Multimodal retrieval ^91^ (graph mining; contract classification)	96	88	91	89	–
Role identification ^94^ (graph mining)	82.35	–	–	–	Purity: 82.35, NMI: 78.34
Community detection (Bitcoin) ^92^ (graph mining)	–	–	–	–	Modularity: 0.37, silhouette: 0.58
Secure lottery design ^93^ (smart contract security)	–	–	–	–	Block time: 3 s, TPS: 41

Third, we critically appraise the evidence base. Using the quality checklist and the extraction matrix, we quantify how often the included studies compared against baselines, controlled for bias or validated their data, released data or code, reported computational cost, and evaluated under open-world or temporal conditions. This appraisal shows where high reported scores do not yet reflect deployability.

Fourth, we identify implementation gaps by systematically uncovering the semantic challenges in detecting localized slang within low-resource languages and the operational disconnect between high-overhead academic models and resource-constrained government infrastructure.

Finally, we derive a research roadmap from these gaps, prioritizing adaptive systems that handle concept drift, lightweight edge deployment, Web3 forensics, and explainable AI (XAI) for legal admissibility. We also release the full screening, quality-assessment, and extraction data so that the synthesis can be replicated and extended into shared benchmarks.

## Methods

This section presents the systematic methodology used to conduct an SLR on an OGDS. ^107^ This review follows the established evidence-based synthesis guidelines for computer science and software engineering from Kitchenham and Charters ^108^ and Carrera-Rivera et al. ^109^ To ensure rigor, reproducibility, and transparency, we organized the process into three phases: Planning, Conducting, and Reporting. This review follows the PRISMA 2020 statement ^110^; the completed PRISMA 2020 checklist and flow diagram are available as reporting guidelines (see Data availability).

Figure 2 provides an overview of the three-phase workflow. The left block corresponds to the planning phase, in which we identified population, intervention, comparison, outcome, and context (PICOC) needs, mapped research questions, and defined the review protocol. The central block depicts the conducting phase, in which we query data sources, filter candidate studies using predefined criteria, and apply quality assessment and data extraction procedures. The right block represents the reporting phase, in which we synthesize and present the extracted evidence. That is, it visually links all the subsequent tables and subsections in this section into a single coherent pipeline.

The following subsections describe each phase in Figure 2 in detail, with explicit references to the tables and diagrams that formalize the protocol’s individual steps.

### Planning

The Planning phase ensured that the review was conceptually sound and methodologically consistent. In Figure 2, this corresponds to the leftmost block and comprises three activities: (i) defining the review scope using the PICOC framework; (ii) formulating the research questions derived from that scope; and (iii) establishing a review protocol that governs the subsequent phases.

### PICOC framework

We adopted the PICOC framework to operationalize the review scope. Table 3 translates the abstract PICOC elements into concrete SLR choices. The first column lists each component and the second column provides an instantiation of the online gambling domain. For example, the population is restricted to online gambling websites or related digital content, whereas the intervention covers computational detection systems, whether rule-based, statistical, machine learning, or deep learning. The comparison item clarifies that traditional rule-based or manual approaches serve as baselines, and the outcome and context entries emphasize performance, robustness, and a computer science perspective. Together, these rows explicitly define the boundaries within which the remainder of the review was conducted. For this review, an online gambling detection system (OGDS) is a computational pipeline that takes platform data (web content, application code or interfaces, social media posts, messages, network traffic, transactions, or user activity logs) and identifies, classifies, clusters, or scores one of four targets: (i) illegal gambling services and their infrastructure, such as websites, domains, applications, and injected or defaced pages; (ii) promotion and distribution channels, such as spam, social media posts, and hidden advertisements; (iii) financial actors and transactions, such as fund accounts and cryptocurrency gambling addresses; and (iv) gambling-related harm and risk behavior detected from platform data. We included studies that proposed, applied, or evaluated such a pipeline with measurable metrics; this broad definition is reflected in the six operational domains in the Results. Target (iv) was retained because operators and regulatory bodies apply computational methods to the same platform data for consumer protection; ^44^ ^,^ ^56^ ^,^ ^63^ studies under this target screen or profile users for risk and are not clinical diagnostic tools (see Discussion).

### Research questions

Based on the gaps identified in the Introduction, specifically data drift and sophisticated obfuscation, we formulated four research questions (RQs; Table 4) to guide the synthesis. RQ1 and RQ2 address how detection systems are used across modalities and integrated with other architectures, whereas RQ3 and RQ4 address implementation challenges and future directions.

### Review protocol

The review protocol links the conceptual layer in Figure 2, Table 3, and Table 4 to the operational procedures used during the conducting phase. It specifies (1) the data sources, that is, the academic databases and digital libraries queried; (2) the search terms and queries, that is, how the Boolean search string is constructed and applied; (3) the selection criteria, that is, the explicit inclusion and exclusion rules used during screening; (4) the quality assessment criteria and scoring scheme used to evaluate methodological rigor; (5) the data extraction attributes extracted from each study and mapped to the RQs; and (6) the synthesis strategy, that is, how quantitative and qualitative evidence is integrated in the analysis.

We later instantiated and quantified these elements using the PRISMA diagram and the tabulated criteria in the Conducting subsection. Defining the protocol before searching reduces selection bias, provides a clear audit trail, and ensures that all counts and criteria reported in the figures and tables are derived from a consistent procedure.

### Conducting

The Conducting phase is shown in the central block of Figure 2. The protocol included four main activities: database search, study selection (including inter-rater reliability assessment), quality assessment, and data extraction.

### Data sources and search string

The SLR targets primary studies on OGDS published in reputable scientific sources. Accordingly, the study queried four major databases: Scopus, ScienceDirect, The Institute of Electrical and Electronics Engineers (IEEE) Xplore, and ACM Digital Library. Together, these sources index the main peer-reviewed venues for computer science and cybersecurity research: IEEE Xplore and the ACM Digital Library cover the major computing conferences and transactions, ScienceDirect covers Elsevier’s computing and security journals, and Scopus adds multidisciplinary coverage across publishers. We did not search health and psychology databases (e.g., PubMed, PsycINFO) because the review context is computer science ( Table 3); we therefore included studies on gambling-related harm only when they were indexed in these sources and applied a computational method. The distribution of included studies by country, venue, and domain reported in the Results reflects the same query and filters applied across these four databases.

The Boolean search string operationalizes the PICOC-driven scope by intersecting terms related to OGDS:

(“online gambling” OR “gambling platform” OR “gambling website” OR “gambling app” OR “illegal gambling”) AND (“identification” OR “detect” OR “detection” OR “system”)

We identified records in IEEE Xplore, the ACM Digital Library, ScienceDirect, and Scopus. Counts correspond to the screening, quality assessment, and final inclusion worksheets of the underlying data. PRISMA, Preferred Reporting Items for Systematic Reviews and Meta-Analyses; QA, quality assessment.

We adapted the query to each database's syntax while preserving the same logical structure. The retrieval counts shown in Figure 3 are the direct outcomes of applying this query. We built the string from two concept blocks. The first block names the object of detection (online gambling platforms, websites, apps, and illegal gambling) rather than individual game types such as betting, casino, slot, or lottery, because these game-specific terms are also used widely in finance, game theory, and game-design research and would retrieve many records unrelated to gambling services. The second block combines detection terms with the broader term “system,” so we also retrieved studies describing an identification or monitoring pipeline without using the word “detection”; we handled the resulting over-retrieval at screening by excluding 24 reports as unrelated to online gambling detection. We deliberately did not add algorithm-specific terms (e.g., machine learning, deep learning, classification) so rule-based, forensic, and statistical systems would not be excluded at the search stage. Local-language terms (e.g., *judi online*) were not used because eligibility was limited to English-language publications.

### Study selection

We conducted database searches on 9 January 2026 to capture the most recent studies. We exported all retrieved records to Parsif.al for deduplication and screening.

Records retrieved from all sources were merged in Parsif.al. As summarized in the PRISMA flow diagram ( Figure 3), the four databases jointly yielded 1,866 records: IEEE Xplore (n = 119), ACM Digital Library (n = 194), ScienceDirect (n = 1,262), and Scopus (n = 291). Database-specific filters (publication year, subject area, document type, and language) removed 1,593 records, leaving 273 records (Scopus, n = 47; ScienceDirect, n = 113; IEEE Xplore, n = 44; ACM Digital Library, n = 69). After removing 20 duplicates, we screened 253 records by title and abstract and excluded 156. Two reviewers independently screened every record at the title-and-abstract stage and every retrieved report at the full-text stage; disagreements were resolved by consensus discussion.

We sought full texts for the remaining 97 reports; we could not retrieve four articles. The other 93 reports were assessed against the inclusion and exclusion criteria in Table 5, and 38 were excluded: topic unrelated to online gambling detection or misuse of the keywords (n = 24), secondary studies (n = 3), primary studies without a detection method or measurable evaluation metrics (n = 5), domain outside computer science without a technical method (n = 5), and non-scientific publications (n = 1). The remaining 55 studies proceeded to quality assessment and then to an independent secondary eligibility assessment by two reviewers.

### Quality assessment

After full-text screening, 55 studies met the inclusion criteria and underwent structured quality assessment (QA) using the 10-item checklist in Table 6, which evaluates methodological rigor and reporting completeness. One reviewer scored each study against the checklist, and a second reviewer verified the scores; disagreements were resolved by consensus. Each criterion was scored 1 (yes) or 0 (no); Q8 (results better than the baseline) was scored 0 when no baseline comparison was reported (N/A). The maximum possible score was 10, and we excluded studies scoring below 5. We used a purpose-built checklist rather than a clinical risk-of-bias tool because the primary studies are engineering evaluations rather than clinical trials or prognostic models; its items target reporting completeness and comparability of evaluation, and it is not a validated risk-of-bias instrument. Because Q8 depends on Q7, a study without a baseline loses two points; we retained this scoring because the absence of a baseline prevents any judgment of whether a proposed method improves detection.

During the QA phase, we excluded one study by Buribayev et al. (2025) ^87^ because its QA score (3) fell below the threshold of 5, leaving 54 studies. One of these, Le Grand (2025) ^64^ (QA score 8), had initially been set aside as a preprint; when verifying the search records, we found that the search had retrieved its journal version, so we reinstated it. The other 53 studies entered the inter-rater agreement analysis. Table 6 presents the QA checklists used in this study.

Table 7 summarizes the distribution of quality scores across the 54 studies that passed quality assessment. The mean QA score across these 54 studies was 7.89, with a median of 8.0 (the 39 studies finally included had a mean of 8.6, range 6–10), indicating moderate to high methodological quality. A total of 22 studies (40.7%) were classified as high quality, whereas 26 studies (48.1%) were rated as moderate quality. Only six studies (11.1%) fell into the low-quality category, all with the minimum accepted score of 5.

These results suggest that most reviewed studies met baseline quality expectations. However, QA alone was not considered sufficient for final inclusion, as domain relevance and architectural contribution were assessed separately in inter-rater agreement.

### Inter-rater reliability

After quality assessment, two reviewers independently judged whether each of the 53 studies met the secondary eligibility criterion described below. They agreed on 49 of 53 studies, including 34 jointly marked for inclusion and 15 jointly marked for exclusion. Disagreements occurred in four cases, in all of which Reviewer A recommended exclusion and Reviewer B recommended inclusion. Table 8 lists the corresponding agreement matrices.

The observed agreement ( $P_{o}$ ) was calculated as:

$$P_{o}=\frac{34+15}{53}=\frac{49}{53}=0.925$$ (1)

The expected agreement by chance ( $P_{e}$ ) was derived from the marginal probabilities of inclusion and exclusion:

$$P_{e}=\left(\frac{34}{53}\times \frac{38}{53}\right)+\left(\frac{19}{53}\times \frac{15}{53}\right)=\frac{1577}{2809}=0.561$$ (2)

Substituting Equations (1) and (2) into Cohen’s kappa formula ^111^ yields:

$$\kappa =\frac{P_{o}-P_{e}}{1-P_{e}}=\frac{0.925-0.561}{1-0.561}=0.83$$ (3)

According to the Landis and Koch interpretation scale, ^112^ a kappa value of 0.83, as shown in Equation (3) and Table 9, indicates almost perfect agreement (0.81–1.00), supporting the reliability of the eligibility decisions.

Following the reliability assessment, the two reviewers resolved the four disagreements through structured consensus discussion, and all four were included. The secondary eligibility criterion that both reviewers applied emphasized technical depth and system-level relevance, focusing on whether a study explicitly contributed to the design, implementation, or evaluation of OGDS. This criterion was not part of the protocol defined before the search; it was added after quality assessment because several studies met the formal criteria and the quality threshold without offering a computational detection component, and it therefore constitutes an amendment to the protocol.

The reinstated study ^64^ was assessed against the secondary criterion jointly by both reviewers after the agreement analysis and was included; it is therefore not part of the kappa calculation. In total, 15 studies (all joint exclusions by both reviewers) were excluded despite acceptable QA scores, primarily because of insufficient architectural detail, relevance limited to policy commentary without a computational or analytic component, or a lack of reproducible technical evaluation. As a result of this two-stage decision process, we retained 39 studies as the final primary studies (34 joint inclusions, four inclusions after consensus, and the reinstated study) for comprehensive data extraction and synthesis.

### Data extraction and synthesis

One reviewer extracted the fields listed in Table 10 from each included study, and a second reviewer verified the extracted data; disagreements were resolved by consensus. This section describes the two complementary stages for transforming the raw extraction matrix into actionable insights, as listed in Table 10. First, we counted the frequency of occurrence of each key element, such as data modalities, domains, tasks, metrics, and architecture. This calculation produces a numerical picture that answers the initial research question. Next, we review the studies to identify common ideas, such as optimization strategies, common ways to combine systems with other architectures, and limitations reported by the authors. We grouped these themes into the following research question. By combining these numbers and themes, we draw conclusions, uncover research gaps, and suggest practical directions for future research on the OGDS.

### Reporting

The reporting phase translates the results of data extraction and synthesis into a transparent, reproducible narrative that addresses each research question sequentially. We presented all findings using structured tables, flowcharts, and summary figures to convey study selection, descriptive statistics, and comparative results. We tabulated key quantitative and performance insights and distilled qualitative themes into clearly labeled subsections highlighting optimization strategies, integration patterns, limitations, and future research directions. We provide supplementary materials in an open repository to facilitate independent verification and subsequent meta-analyses.

To ensure transparency and reproducibility, we provided a complete inventory of primary studies. This inventory, together with the screening decisions, quality assessment scores, and data extraction matrix, is openly available under a CC-BY 4.0 license, together with the files used to generate Figure 5.

## Results

### Characteristics of the included studies

This section describes the temporal evolution, publication characteristics, and thematic structure of the 39 included studies. Figure 4a shows that the included studies are unevenly distributed over time: three were published in 2021, seven in 2022, four in 2023, 10 in 2024, and 15 in 2025, so 25 of the 39 studies (64%) appeared in the last two years. These counts describe studies that met our eligibility and quality criteria in four databases rather than total research output, and the annual numbers are small; the decrease from seven studies in 2022 to four in 2023, for example, is a difference of three studies and may reflect sampling variation rather than a change in research activity.

The higher counts in 2024 and 2025 are consistent with growing research interest in OGDS. They coincide with the spread of mobile-first gambling platforms, cryptocurrency payment channels, and socially mediated promotion described in the Introduction, but this review was not designed to test whether these developments caused the increase. The 2025 count may also be underestimated because some late-2025 publications may not yet have been indexed by the search date (9 January 2026).

Figure 4b shows that conference papers (20 studies, 51%) and journal articles (19 studies, 49%) are almost equally represented. We did not compare the design or rigor of the two publication types, so this split should not be read as evidence that conference papers introduce early-stage pipelines while journal articles provide fuller validation. Regardless of venue, only 12 of the 39 studies released code or data ( Table 19), which limits independent verification of the reported results.

Noun phrases were extracted from titles and abstracts and counted once per study (binary counting); the 35 terms that occur in at least three studies are shown. Node size indicates the number of studies containing the term, links indicate co-occurrence in the same title or abstract, and colors indicate the five clusters computed by VOSviewer. VOSviewer does not display labels that would overlap (here, user, next to identification).

Figure 5 shows the term co-occurrence map of the 39 included studies, created in VOSviewer Online version 1.2.4. ^113^ The terms are noun phrases extracted from the titles and abstracts with part-of-speech tagging (NLTK 3.10); plural nouns were reduced to the singular, spelling variants and abbreviations were merged and generic research terms were removed with a VOSviewer thesaurus file, and each term was counted once per study (binary counting), so node size indicates the number of studies in which a term appears. Terms occurring in at least three studies were retained (35 terms and 284 co-occurrence links). VOSviewer normalized the links by association strength ^114^ and computed the layout (attraction 2, repulsion 1) and the clustering (resolution 1), all at their default values, which yielded five clusters; no term, link, position, or cluster was edited manually. The extraction script, thesaurus, input network, and VOSviewer map file are deposited with the underlying data. The most frequent terms are gambling (14 studies), detection (11), gambling website and website (10 each), and accuracy, online gambling, and recall (8 each).

Read descriptively, the clusters group (1) website and gambling website with website-content terms (URL, image, text, textual content, and semantic feature) and society; (2) detection and identification with user, user activity, community, app, threat, and TF-IDF; (3) evaluation metrics (accuracy, precision, and recall) with machine learning, promotion, service, attack, and Indonesia; (4) gambling, gambler, and gambling behavior with security, internet, pornography, and blockchain technology; and (5) online gambling and online gambling activity with domain and government. App and blockchain technology each occur in only three studies, and methods such as deep learning and graph neural networks did not reach the threshold as standalone terms, partly because they usually appear within longer noun phrases (e.g., multi-view graph neural network framework) that are counted as separate terms. Because the map rests on 39 studies and on how authors phrase their titles and abstracts, these patterns indicate emphasis within this corpus; they do not establish the thematic structure of the wider field.

### RQ1: How has the system been used and utilized in various modalities and tasks of online gambling detection?

Figure 6 and Table 11 illustrate OGDS deployment across heterogeneous application domains and analytical objectives. The figure provides a high-level conceptual mapping between the operational domains and analytical techniques, whereas the table enumerates concrete implementations and representative studies. These visual and tabular summaries show that the literature is strongly oriented towards automated identification, filtering, and risk assessment of illegal gambling activities across web platforms, mobile ecosystems, communication channels, financial infrastructure, and network layers.

Rather than a single analytical paradigm, system use is shaped by the interaction among data characteristics, platform constraints, and enforcement objectives. Website-oriented studies prioritize scalable inspection of content and traffic artifacts, mobile-focused work emphasizes structural and interaction-level analysis, and social media–centric research focuses on linguistic patterns and dissemination dynamics. In parallel, harm detection and blockchain-oriented studies extend detection beyond content identification to behavioral inference and ecosystem-level risk analysis. This diversity reflects a gradual expansion of research goals from surface-level content recognition to deeper modeling of user behavior, financial flows, and infrastructural dependencies.

To contextualize these usage patterns, Figure 7 shows the geographic distribution of the 39 primary studies included in this review. A pronounced concentration was observed in China (18 studies), followed by Indonesia (7), and the United States (4), with smaller contributions from South Korea (2) and Thailand (2), and isolated studies from India, Switzerland, Russia, the Philippines, Portugal, and Canada. This uneven distribution is not merely descriptive; it also affects the design, evaluation, and validation of detection systems.

Studies from China tend to address large-scale, highly adaptive gambling ecosystems spanning mobile platforms, encrypted traffic, and cryptocurrency infrastructure. Consequently, they often use graph-centric representations, network-aware learning, and cross-layer analysis to capture complex operational dependencies. In contrast, research from Indonesia and other Asian countries often targets rapidly proliferating promotional content on websites, social media, and messaging services. These studies typically emphasize deployability, interpretability, and compliance with local regulatory and resource constraints, leading to a stronger focus on content-based inspection and rule-guided automation. The United States and Europe’s contributions, while fewer, are more prominent in harm detection and responsible gambling research, reflecting regulatory priorities focused on consumer protection rather than outright prohibitions.

Taken together, Figure 6, Figure 7, and Table 11 show that OGDS use is inherently task-specific and regionally conditioned. Platform dominance, legal frameworks, data accessibility, and computational resources influence methodological choices. Consequently, RQ1 reveals that current systems are optimized for localized threat models and enforcement contexts, rather than for globally standardized deployment. This finding underscores the importance of cross-regional benchmarking and developing more diverse datasets to support broader generalization in future research.

### Website detection

Website detection is the most extensively studied domain, encompassing the identification of gambling websites, defacement attacks on institutional domains, and so-called “fake-normal” sites designed to evade filtering mechanisms. The literature reflects a layered evolution from lightweight inspection techniques to more adaptive, structurally informed analyses.

Early, operationally efficient approaches relied on signature- and rule-based mechanisms. Harahap and Ridho ^2^ employed a Suricata IDS with keyword-based signatures to detect intrusions in the Indonesian academic domains (.ac.id). Erawan et al. ^3^ developed a bash script that leverages real-time inotify monitoring to identify file injections associated with gambling content on government websites (.go.id). Teppap et al. ^60^ focused on content extraction, using BeautifulSoup to uncover hidden gambling-related material embedded within web search results.

As gambling sites adopt more dynamic and evasive strategies, learning-based methods have emerged to capture patterns beyond static rules. Thianphan and Jearanaitanakij ^78^ applied a Hybrid Ensemble for website classification, whereas Wang et al. ^49^ exploited co-training to leverage unlabeled data. In a forensic context, Kurniawan and Triayudi ^74^ combined Wazuh and Velociraptor to reconstruct web defacement incidents. Feng et al. ^73^ and Zhang et al. ^72^ shifted their focus to POST traffic clustering and user behavior modeling, enabling the identification of site templates and betting patterns that are resilient to superficial content changes.

Several studies have integrated visual, textual, and structural cues to counteract deliberate obfuscation. Wen et al. ^62^ proposed MEDAL for data augmentation across heterogeneous website representations, whereas Zhang et al. ^77^ introduced DRSDetector by combining domain-level and semantic features. Graph-based formulations are particularly prominent. Ma et al. ^52^ ^,^ ^76^ employed HGNN to detect fake-normal sites and addressed imperfect datasets through lightweight graph constructions. Wang et al. ^50^ ^,^ ^59^ further demonstrated that vision–language fusion models can reveal gambling content intentionally hidden in images rather than HTML text.

Beyond content inspection, researchers also considered financial aspects. Zheng et al. ^6^ extended website detection to financial forensics by developing a YOLOv4-based system to automatically extract illegal fund accounts directly from gambling site interfaces.

### Mobile app

Mobile app detection focuses on identifying illegal gambling applications distributed through unofficial channels, often disguised or repackaged to evade app store policies. A defining characteristic of this domain is the emphasis on structural relationships rather than surface content.

Lu et al. ^53^ introduced TacDroid, which models application behavior through UTG and captures navigation patterns indicative of gambling functionality. Chen et al. ^58^ adopted a similar UTG-based strategy in DeUEDroid to reinforce the effectiveness of UI-level representations. Gu et al. ^51^ proposed a complementary approach using HeCGamb, constructing a heterogeneous graph over encrypted traffic to identify gambling apps without decrypting payloads and addressing privacy and encryption constraints.

### Social media and spam messages

This domain addresses the dissemination of gambling promotions through public platforms and private messaging channels. Linguistic variability, high message volume, and rapid content mutation are central challenges.

Liem et al. ^47^ and Santika et al. ^54^ leveraged IndoBERT to detect gambling promotions in YouTube comments and Indonesian social media, including semi-supervised extensions to handle limited labeled data. Wang et al. ^4^ conducted a large-scale analysis of illegal goods promotions on Twitter. In contrast, Lerma et al. ^85^ applied Tau-U statistics to examine the relationship between user activity and exposure to gambling content.

For SMS-based campaigns, Min et al. ^48^ developed a honeypot system using a Readable Transformation Technique (RTT) to decode obfuscated gambling URLs. Their follow-up study ^57^ refined this approach for illegal online gambling detection, with both studies applying learning-based classifiers to distinguish gambling-related spam from benign messages.

### Harm detection

Unlike the other domains, which classify services, channels, or transactions, studies in this domain score or group individual gamblers or populations, and their reference standards are proxies rather than clinical diagnoses. Harm detection shifts the focus from identifying illegal platforms to protecting users through behavioral and risk assessments. Stechschulte et al. ^56^ performed in-database feature extraction for early risk identification, whereas Murch et al. ^63^ evaluated the temporal stability of detection models. Ghaharian et al. ^45^ clustered users based on payment transactions to reveal high-risk behavioral profiles. Le Grand ^64^ combined an XGBoost classifier and SHAP feature attribution with a large language model (LLM)-based retrieval-augmented generation (RAG) pipeline that produces an individualized, human-readable justification for each addiction-risk classification.

Researchers also explored social and psychological dimensions. Antonio et al. ^46^ employed a UTAUT3-based survey to assess gambling intentions, Ling et al. ^83^ analyzed psychometric networks related to loot-box risk, and van der Maas et al. ^84^ used interrupted time series analysis on Reddit discussions to study the impact of gambling legalization. In predictive settings, Vatian et al. ^86^ applied hybrid neural networks to churn prediction, whereas Peres et al. ^44^ used time-series clustering to identify behavioral trajectories associated with potentially problematic gambling.

### Crypto and blockchain

This domain examines gambling activities embedded in decentralized ecosystems. Huang et al. ^91^ employed retrieval techniques to track crypto gamblers, Wang et al. ^94^ identified hidden operational roles within transaction graphs, and Wu et al. ^92^ applied community detection to blockchain-based social networks. From a preventive perspective, Datta et al. ^93^ designed a secure and fair lottery protocol using smart contracts on the Binance Smart Chain.

### Infrastructure

Infrastructure-level studies have focused on mitigation and communication security. Kuntoro et al. ^1^ optimized ISP-level blocking through DNS response policy zone (RPZ) clustering, whereas Ghani et al. ^75^ applied Isolation Forest and Random Forest models to detect and mitigate DDoS attacks and backdoors associated with gambling operations.

### Modalities and datasets used

Research on OGDS is characterized by a highly fragmented dataset landscape, with each study constructing bespoke datasets aligned with its operational context, regulatory environment, and platform accessibility. Apart from two studies by the same group that used the same KISA SMS corpus, ^48^ ^,^ ^57^ no dataset was reused across primary studies, making performance claims inherently context-dependent.

Textual datasets dominate content-centric detection. For example, ^2^ used 1,200 defaced and 1,200 benign HTML pages from Indonesian academic domains, while ^60^ analyzed keyword patterns in crawled Thai university websites. SMS datasets are central to spam detection, as shown in, ^48^ ^,^ ^57^ both of which rely on 30,527 real-world messages from the Korean Internet & Security Agency (KISA). Social media corpora range from 12 million posts and 580,000 accounts ^4^ to smaller, platform-specific collections, such as the 10,101 YouTube comments in, ^54^ which are acknowledged to be vulnerable to linguistic drift. ^47^

More complex modality datasets combine multiple information sources. Studies such as ^50^ ^,^ ^62^ integrated website screenshots, OCR-extracted text, and HTML content, reflecting the empirical observation that gambling operators increasingly conceal semantics in images. Behavioral and financial datasets underpin harm detection research, including transaction logs from U.S., ^45^ Swiss, ^56^ and Portuguese ^44^ operators, offering high ecological validity but limited reproducibility.

Graph-structured datasets appear in mobile app studies, ^53^ ^,^ ^58^ encrypted traffic analysis, ^51^ and blockchain investigations. ^91^ ^,^ ^94^ Survey-based and policy-oriented datasets were used to analyze gambling intentions and the effects of policy changes. ^46^ ^,^ ^84^

Three patterns were evident across all studies. First, the modality datasets were bespoke and non-reusable. Second, data characteristics are tightly aligned with research objectives, from content inspection to behavioral inference. Third, the absence of shared benchmarks fundamentally limits comparability. Therefore, progress in OGDS is constrained less by algorithmic capability than by the lack of standardized, longitudinal, cross-regional datasets that reflect real-world adversarial dynamics.

### RQ2: How can existing systems be integrated with other architectures to improve performance in online gambling detection tasks?

Integrating detection systems with complementary architectures has become a fundamental strategy for overcoming the limitations of a single model, particularly amid dynamic obfuscation techniques and evolving gambling infrastructure. ^50^ ^,^ ^51^ ^,^ ^62^ Based on an analysis of the 39 primary studies, we visually mapped the system integration patterns, as shown in Figure 8.

Figure 8 illustrates how various data modalities (text, visual, graph, and behavior) are processed through different integration layers to produce a precise detection output. Taxonomically, Table 12 classifies these approaches into six categories: multimodal feature fusion systems, graph-structural integration systems, hybrid ensemble learning systems, semantic systems, harm systems, and rule-based operational systems.

### Multimodal feature fusion systems

This architecture addresses the “semantic gap,” where gambling operators hide text keywords in images or use complex HTML structures. Wang et al. ^50^ proposed a hybrid method that fine-tuned ResNet34 for visual features and Bi-LSTM for text, achieving an F1-score of 99.31%. Wen et al. ^62^ applied a similar approach via the MEDAL framework, which uses tri-training to integrate visual (VGG16) and textual classifiers. Wang et al. ^49^ applied a co-training strategy to screen for visual capture and convert it into text using OCR. Zhang et al. ^77^ (DRSDetector) combined Transformer-encoded domain character features, LightGBM-learned resource features, and HAN-based semantic features. Wang et al. ^59^ utilized a vision-and-language model with sparse multi-head attention for efficiency in long texts. Ma et al. ^76^ used a lightweight graph approach that combines structural and textual features, whereas Huang et al. ^91^ used a multimodal retrieval method to identify crypto gamblers.

### Graph-structural integration systems

Recognizing that gambling applications and sites often have unique navigation structures or transaction networks, this architecture integrates content analysis with graph modeling. TacDroid by Lu et al. ^53^ and DeUEDroid by Chen et al. ^58^ combined static and dynamic analyses to build a UTG that effectively detects illegal applications that hide their true behavior. At the network level, Gu et al. ^51^ (HeCGamb) model relationships among encrypted traffic flows as a heterogeneous graph, and Ma et al. ^52^ use an HGNN to model relationships among websites through hyperlinks, title and meta terms, and IP addresses. Wu et al. ^92^ applied community detection to blockchain social networks, Wang et al. ^94^ identified hidden roles (such as delegates) in crypto casinos using graph analysis, and Zhang et al. ^72^ grouped user behaviors based on the site navigation structure.

### Hybrid ensemble learning systems

The system integrates several machine learning algorithms to balance computational efficiency and detection accuracy. Ghani et al. ^75^ combined an Isolation Forest for anomaly detection (pre-filtering) with a random forest (RF) for precise network attack classification. Thianphan and Jearanaitanakij ^78^ used a Hybrid Ensemble that stacks the URL and content classifiers to overcome the weaknesses of single features. Vatian et al. ^86^ combined a convolutional neural network (CNN), long short-term memory (LSTM), and graph neural network (GNN) into a single hybrid model for high-accuracy customer churn prediction. Feng et al. ^73^ optimized support vector machine (SVM) parameters using particle swarm optimization (PSO) to classify POST messages.

### Semantic systems

Recent studies have integrated pre-trained transformer models to handle linguistic complexity, slang, and local contexts. Liem et al. ^47^ found IndoBERT to be the most effective of several deep and machine learning models for detecting Indonesian online gambling promotion, and Santika et al. ^54^ applied semi-supervised IndoBERT to Indonesian YouTube comments; both reported accuracies above 98%. Wang et al. ^4^ used a multilingual language model to detect illegal goods promotion on Twitter. Min et al. ^48^ ^,^ ^57^ applied natural language processing (NLP) techniques to SMS spams to extract and decode obfuscated gambling URLs.

### Harm systems

This pattern integrates longitudinal data analysis to estimate the risk of gambling-related harm and to profile players. Stechschulte et al. ^56^ used in-database feature extraction to detect problem gambling behavior early, whereas Peres et al. ^44^ applied time-series clustering to distinguish regular players from groups with potentially problematic gambling patterns. Ghaharian et al. ^45^ extend this analysis to financial aspects by identifying risk markers using payment transaction ratios. Murch et al. ^63^ evaluated the temporal stability of ML models in detecting gambling harm (harm detection). Le Grand ^64^ paired an XGBoost risk classifier with an LLM-based RAG pipeline that explains each classification. Ling et al. ^83^ used network analysis to link loot-box behavior to gambling disorders, whereas Antonio et al. ^46^ used an extended UTAUT3 model to analyze behavioral intentions. Lerma et al. ^85^ and van der Maas et al. ^84^ analyzed user activities and discussion forums to understand the impact of gambling promotions and legalization of sports betting.

### Rule-based and operational systems

This architecture integrates detection logic with operational tools by bridging the gap between algorithmic detection and practical mitigation. Harahap and Ridho ^2^ and Kurniawan and Triayudi ^74^ integrated an IDS (Suricata/Wazuh) with real-time notifications via a Telegram Bot. Erawan et al. ^3^ used a lightweight bash script with inotify to quarantine file injections at government sites. Kuntoro et al. ^1^ optimized the domain blocking at the ISP level using DNS RPZ clustering. Other operational systems include extracting illegal fund accounts (Zheng et al. ^6^), hidden advertising detection with BeautifulSoup (Teppap et al. ^60^), and a smart contract-based fair lottery system (Datta et al. ^93^).

A synthesis across studies has shown that OGDS are rarely applied in isolation. Instead, researchers have systematically hybridized the detection core with complementary architectural motifs to balance context modeling, texture fidelity, and computational efficiency. We evaluated these cross-cutting themes quantitatively using a series of performance metrics across various application domains.

### Performance analysis across operational domains

In the website detection domain, high accuracy competes with computational efficiency. Table 13 compares rule-based approaches, traditional machine learning, and complex multimodal systems.

The values in Table 13 suggest that multimodal systems, such as MEDAL and Hybrid Fusion, counter content obfuscation by integrating visual and textual features, although the values come from different datasets. In contrast, for resource-constrained environments, rule-based approaches such as bash scripting offer high efficiency, with memory usage of only 20 MB.

In the mobile application domain, graph-based approaches reported higher detection performance than conventional static analysis in detecting illegal applications that hide their true behavior ( Table 14). HeCGamb reports perfect precision (100%) by modeling encrypted traffic as a heterogeneous graph, which suggests that relationships between traffic flows can be informative even when payloads are encrypted; in its open-world evaluation, performance decreased to an F1-score of 94.1% and an AUC of 96.8%. ^51^

For text, LLMs dominate context understanding, especially in social media and instant messaging, and Table 15 compares the effectiveness of the various NLP and spam detection techniques. For short texts such as SMS, the RTT technique is crucial for decoding obfuscated URLs, achieving 99.77% accuracy. IndoBERT-based models reported F1-scores of 97.24% ^47^ and 98.50% ^54^ on Indonesian gambling-promotion text; because these values come from different datasets, they indicate strong within-study performance on slang-rich text rather than general superiority.

Focusing on user behavior reveals unique challenges in the evolution of gambling patterns over time; Table 16 presents the evaluation metrics for the behavioral, risk, and psychometric analyses. Murch et al. ^63^ found that all ten indicators of problematic online gambling shifted significantly between 2019 and 2022 and that the area under the precision–recall curve of two previously validated models changed significantly (for PGSI 8+, ΔAUPRC = +7.06%; t = 7.21, p < 0.001); recalibrating the decision thresholds realigned classification performance with the originally validated levels, indicating the need for periodic recalibration. Vatian et al. ^86^ reported an AUC of 94% with a hybrid CNN + LSTM+GNN model that combines temporal features and interaction patterns; however, this result concerns churn prediction in web services and therefore does not demonstrate that drift in gambling-harm detection is mitigated.

Values are as reported in each primary study. Metrics quantify agreement with each study’s own reference standard (exclusion lists, self-report screening scores, refined labels, or internal cluster indices) and are not measures of clinical diagnostic accuracy.

Finally, the crypto and network infrastructure domains emphasize the importance of structural analysis and large-scale operational efficiency; Table 17 and Table 18 summarize system performance in decentralized ecosystems and ISP-level mitigation, respectively. Low-level optimization, such as DNS clustering, is crucial for ISPs that handle millions of blacklist lines. Collectively, comparisons between these tables suggest that no single architecture suits every domain: multimodal systems and transformers excel in content accuracy, whereas graph-based models are essential for blockchain and application structure analyses.

**Table 18.** Reported performance of methods in the infrastructure domain.

Method (task)	Time/speed	Acc (%)	Prec (%)	Outcome
DNS RPZ clustering ^1^	31.73 ms	–	–	Speed improvement: ~10%
AI-driven network security ^75^	–	93	88 (DDoS class)	DDoS and backdoor mitigation

Several studies support research transparency by providing source code or publicly available datasets, and Table 19 summarizes these resources to facilitate replication and further development. Despite these contributions, the current OGDS landscape remains constrained by linguistic barriers, except for studies on large-scale Twitter analysis ^4^ in the Social Media domain that use a multilingual text dataset. Most available datasets are strictly localized to their respective regional languages, and the lack of standardized, cross-linguistic, and universally accessible benchmarks limits the research community’s ability to perform large-scale comparative evaluations across diverse adversarial contexts.

**Table 19.** Primary studies with publicly available code or datasets.

Domain	Task	Method	Resource link
Website detection	Multimodal	HGNN ^52^	https://github.com/stunstu/mal-multi/tree/main/dataset
Website detection	Financial forensic	Illegal fund analysis ^6^	https://www.kaggle.com/datasets/shenaozheng/ui-detection-for-gambling-sites
Mobile app	Graph-based detection	TacDroid (UTG) ^53^	https://github.com/initfunction/TacDroid
Mobile app	Graph-based detection	DeUEDroid ^58^	https://github.com/HypoopyH/DeUEDroid
Mobile app	Graph-based detection	HeCGamb ^51^	CSTNET-300: https://ieee-dataport.org/documents/cstnet
Social media and spam messages	NLP and deep learning	Twitter analysis ^4^	https://illicit-promotion.github.io/
Social media and spam messages	NLP and deep learning	IndoBERT ^47^	https://github.com/tyogoutomo/ INDONESIAN_ONLINE_ GAMBLING_WEBSITE_PROMOTION_DATASET
Social media and spam messages	NLP and deep learning	IndoBERT ^54^	https://doi.org/10.5281/zenodo.15424844
Harm detection	Behavioral analysis	Transaction clustering ^45^	https://doi.org/10.17632/9j5gcygnwg.1
Harm detection	Risk analysis	Explainable XGBoost with LLM-based RAG ^64^	https://github.com/jermaine-le-grand/Online-Gambling-Addiction-Modeling
Crypto and blockchain	Graph mining	Multimodal retrieval ^91^	https://github.com/AwesomeHuang/Bitcoin-Gambling-Dataset
Crypto and blockchain	Graph mining	Community detection ^92^	ELTE Bitcoin: http://vo.elte.hu/bitcoin

*Note*: HGNN, heterogeneous graph neural network; LLM, large language model; NLP, natural language processing; RAG, retrieval-augmented generation; UTG, user interface transition graph; XGBoost, extreme gradient boosting.

### RQ3: What are the main challenges and limitations associated with implementing online gambling detection systems?

Despite rapid advances in detection architectures, deploying OGDS in practice faces obstacles that are adversarial and operational as well as algorithmic. We grouped the challenges reported across the 39 studies into four dimensions, namely data quality and temporal instability, adversarial evasion and obfuscation, semantic and contextual ambiguity, and resource and computational constraints; Table 20 summarizes them with the mitigations proposed in the primary studies, and the following subsections present the evidence for each dimension.

**Table 20.** Main challenges and limitations reported for online gambling detection systems.

Challenge	Key limitations and issues	Potential implementation	Primary sources
**Data quality and temporal instability**	Data drift phenomena (behavioral shifts over time), extreme class imbalance (a target group of 3.65% in one harm-detection study), and scarcity of labeled ground truth	Semi-supervised learning (tri-training), continuous recalibration of thresholds, and multi-stage clustering	^44^ ^,^ ^45^ ^,^ ^56^ ^,^ ^62^ ^,^ ^63^ ^,^ ^72^ ^,^ ^76^ ^,^ ^83^ ^,^ ^84^ ^,^ ^86^
**Adversarial evasion and obfuscation**	Evasion tactics such as hiding text in images, use of WebViews (found in 53% of gambling apps ^53^), traffic encryption, and web defacement on.go.id domains	Multimodal fusion (OCR + visual), dynamic UTG, and HGNN	^2^ ^,^ ^4^ ^,^ ^6^ ^,^ ^48^ ^–^ ^53^ ^,^ ^57^ ^,^ ^58^ ^,^ ^59^ ^,^ ^60^ ^,^ ^73^ ^,^ ^74^ ^,^ ^77^ ^,^ ^78^ ^,^ ^85^
**Semantic and contextual ambiguity**	Difficulty in understanding localized slang (“ *gacor*”, “ *jepe*”), linguistic drift, and actor anonymity in blockchain networks	Transformer-based NLP (IndoBERT), multi-view graph learning, and behavioral role inference	^46^ ^,^ ^47^ ^,^ ^54^ ^,^ ^91^ ^,^ ^92^ ^,^ ^94^
**Resource and computational constraints**	Low-specification government servers, high latency from massive 711 MiB blocklists, and high VRAM costs for visual models	Lightweight Bash scripts (<20 MB memory), DNS RPZ clustering, and lightweight graph scanning	^1^ ^,^ ^3^ ^,^ ^75^ ^,^ ^76^ ^,^ ^93^

*Note*: DNS, domain name system; HGNN, heterogeneous graph neural network; NLP, natural language processing; OCR, optical character recognition; RPZ, response policy zone; UTG, user interface transition graph; VRAM, video random access memory.

### Data quality and temporal instability: challenges to long-term validity

A critical barrier to robust detection is that gambling data change over time, so model validity can decay quickly. Gambling behavior shifts over time (“concept drift”), which changes the performance of previously validated models: Murch et al. ^63^ showed that behavioral indicators shifted significantly within three years and that decision thresholds had to be recalibrated to restore the originally validated classification performance, and Peres et al. ^44^ modeled betting activity as evolving time series. Imperfect and imbalanced data compound the problem at both the service and the user level: Ma et al. ^76^ designed a website detector for sparse, mismatched, and imbalanced training data, and in Stechschulte et al. ^56^ the target group formed only 3.65% of the sample. Wen et al. ^62^ noted the high cost of obtaining quality labels, which led them to adopt semi-supervised tri-training. Behavior adds further complexity: Zhang et al. ^72^ found that users’ operational behaviors on gambling websites required a secondary clustering stage within each template cluster, and Ghaharian et al. ^45^ similarly grouped gamblers by payment-transaction patterns.

### Adversarial evasion and obfuscation: active countermeasures

Gambling operators employ various tactics to evade automated detectors. They hide promotional keywords (e.g., “bet,” “win”) within images to defeat text-based classification; to counter this tactic, Wang et al. ^50^ ^,^ ^59^ and Wen et al. ^62^ demonstrated the importance of integrating visual features, Zhang et al. ^77^ added website resource features, and Thianphan and Jearanaitanakij ^78^ combined URL and textual features. Apps are hardened against static analysis: Lu et al. ^53^ found that 53% of gambling apps (and 76% of pornographic apps) rely on WebView to load dynamic content, which limits the effectiveness of static analysis. Attackers also deface. go.id and.ac.id domains ^2^ ^,^ ^74^ and manipulate social media algorithms to increase content exposure. ^4^ ^,^ ^85^ In addition, special characters in URLs, ^48^ ^,^ ^57^ similar POST message templates, ^49^ ^,^ ^73^ hidden ads, ^60^ illegal account extraction through the UI, ^6^ and TLS traffic encryption ^51^ make deep packet inspection ineffective.

### Semantic and contextual ambiguity: language and anonymity

Cultural nuances and network decentralization create interpretive barriers for automated detection systems. Local slang (such as “gacor” or “jepe”) and deliberate misspellings on Indonesian social media challenge static keyword filters, as identified by Liem et al. ^47^ and Santika et al. ^54^ On blockchains, the anonymity of addresses makes it difficult to distinguish between genuine gamblers, intermediaries (delegates), and bots, a task complicated by heavy noise interference in community observation models ^92^ and by the need for multimodal data collection to track actor identities in Ethereum and TRON networks. ^91^ ^,^ ^94^ Ambiguity also arises from individually varying psychological triggers, where factors such as hedonic motivation and social influence are more dominant than self-control in determining gambling intentions. ^46^

### Resource and computational constraints: barriers to implementation

Accurate systems are often too heavy for operational infrastructure. Government servers are often low-specification, making lightweight Bash scripts (<20 MB) more practical than complex AI models. ^3^ Massive blocklists (711 MiB) cause substantial DNS latency, ^1^ and the computational cost of visual features is high. ^76^ The integration of university network security ^75^ and the use of Binance Smart Chain (BSC) at 41 TPS ^93^ further illustrate the need for solutions that balance mitigation speed and detection accuracy.

In summary, the implementation of the OGDS is hindered by the multidimensional landscape of technical and operational barriers. Evidence from primary studies indicates that detection is not merely a static algorithmic task but a dynamic “arms race” against highly adaptive adversarial tactics such as multimodal hiding in images, WebView-based app hardening, and traffic encryption. Beyond adversarial countermeasures, concept drift and extreme class imbalance threaten the long-term validity of these systems, necessitating continuous model recalibration and semi-supervised learning to mitigate data scarcity.

Furthermore, the transition from academically robust models to real-world deployment reveals a critical trade-off between the detection depth and operational efficiency. Highly accurate visual and deep learning models often incur computational overhead that is impractical for low-specification government infrastructures or high-throughput ISP environments, driving a strategic shift toward lightweight solutions and optimized filtering protocols. Finally, persistent semantic ambiguity in localized slang and the inherent anonymity of blockchain networks underscore the need for more context-aware, multi-view analytical frameworks. Collectively, these findings suggest that the future of effective OGDS depends on computationally efficient architectures that are adversarially resilient and can adapt to shifting user behaviors and evolving infrastructural constraints.

### RQ4: What are the emerging trends and future directions for online gambling detection systems?

Building on the limitations identified in RQ3 and the integration patterns in RQ2, the 39 studies point toward detection systems that are more adaptive, decentralized, and explainable, moving from binary classification (“gambling vs. non-gambling”) toward analysis of the industry chain, actor roles, and adaptive operator behavior. Figure 9 maps these trends from reactive monitoring toward proactive forensic analysis, and Table 21 organizes the corresponding research directions into five pillars that address the gaps identified across the 39 studies and are discussed in the following subsections.

**Table 21.** Future research roadmap for online gambling detection systems.

Research area	Description	Why it matters	Possible methodologies
**Adaptive and automated response**	Systems capable of autonomous recalibration and active data harvesting	Counteracts the rapid concept drift and interface changes that render static models obsolete	Unsupervised template discovery, ^72^ ^,^ ^73^ autonomous threshold recalibration, ^44^ ^,^ ^45^ ^,^ ^63^ semi-supervised tri-training, ^62^ co-training for unlabeled data, ^49^ and automated investigative script generation ^6^ ^,^ ^53^ ^,^ ^60^ ^,^ ^86^
**Deep semantic and entity extraction**	Granular identification of specific operators and slang-heavy communication	Transforms detection from broad filtering to targeted forensic intelligence for law enforcement	NER for contact extraction, ^4,^ ^,^ ^54^ multimodal vision–language models, ^50^ ^,^ ^59^ slang-tuned NLP models, ^47^ longitudinal exposure analysis, ^84^ ^,^ ^85^ and semantic URL decoding ^48^ ^,^ ^57^ ^,^ ^77^
**Web3 and blockchain forensics**	Forensic analysis of decentralized platforms and actor role identification	De-anonymizes participants in environments where traditional URL-based detection is ineffective	Multi-view GNN for role identification, ^94^ multimodal retrieval for crypto gamblers, ^91^ graph noise reduction for community detection, ^92^ and quantum-resistant lottery signing ^93^
**Explainability and interpretability (XAI)**	Development of transparent models that justify detection decisions	Essential for legal admissibility of digital evidence and human operator trust	Relation-based heterogeneous graph modeling, ^51^ multivariate decision model interpretability, ^56^ UI transition structure analysis, ^58^ behavior-based clustering transparency, ^72^ and SHAP-grounded LLM-generated explanations ^64^
**Lightweight and edge AI**	Resource-efficient models for deployment on infrastructure hardware	Enables national-scale mitigation without network latency or high hardware costs	Optimized DNS clustering (RPZ), ^1^ lightweight IDS rules ^2^ and Bash monitoring scripts (<20 MB), ^3^ anomaly detection pre-filtering, ^75^ real-time cloud forensics, ^74^ and resource-optimized semi-supervised graphs ^76^

### Adaptive and automated response: addressing rapid evolution

A major weakness of current systems is model degradation from temporal drift, where behavioral shifts and interface changes render static models obsolete, so future research should prioritize systems that can autonomously recalibrate and actively harvest data. Murch et al., ^63^ Peres et al., ^44^ and Ghaharian et al. ^45^ emphasized the need for models that automatically recalibrate decision thresholds and predictive indicators as player behavioral distributions shift over time. For zero-day template discovery, Feng et al., ^73^ Zhang et al., ^72^ and Lu et al. ^53^ suggested unsupervised clustering and graph autoencoders (GAE) to identify previously unseen gambling site templates and illicit app behaviors, thereby reducing reliance on manual labeling.

Zheng et al. ^6^ and Teppap et al. ^60^ moved toward active investigation, in which systems automatically generate registration scripts and use web scraping to continuously harvest illegal fund accounts and hidden advertising content as operator interfaces evolve. Co-training, ^49^ tri-training, ^62^ and hybrid neural networks ^86^ could further allow future systems to exploit the large amount of unlabeled data available.

### Deep semantic and entity extraction: target precision

Current language models perform well at binary classification but lack the granularity required for actionable forensic intelligence. Santika et al., ^54^ Liem et al., ^47^ and Wang et al. ^4^ highlighted the need to integrate named entity recognition (NER) to extract specific entities, such as operator identities or contact numbers (WhatsApp/Telegram), from slang-heavy social media and Twitter posts. Vision-language models and specialized NLP techniques are needed to handle OCR errors, intentionally distorted text in promotional images, ^49^ ^,^ ^50^ and URL obfuscation in SMS spam. ^48^ ^,^ ^57^ Addressing the impact of social media algorithms ^85^ and policy changes ^84^ also requires longitudinal analysis of user discussions and risky behaviors, such as those found in loot-box ecosystems. ^83^

### Next-generation blockchain forensics: security in the Web3 era

As gambling migrates to decentralized ecosystems, conventional content-based detection becomes secondary to transaction-layer forensics. Datta et al. ^93^ identified that future blockchain-based lottery systems must adopt lightweight signing mechanisms that are resistant to quantum computing threats to ensure long-term integrity, and Wang et al., ^94^ Wu et al., ^92^ and Huang et al. ^91^ advocated multi-view graph learning and modified community detection to distinguish genuine gamblers, automated bots, and intermediary delegates within smart contract networks.

### Explainability and interpretability (XAI): legal trust

For law enforcement to adopt automated systems, models must move away from “black-box” logic toward decision transparency to provide admissible evidence. Gu et al. ^51^ (HeCGamb) and Chen et al. ^58^ (DeUEDroid) demonstrated that visualizing attention weights and UI transition similarities helps explain why a node or application is flagged. Future work could build on multivariate decision models, ^56^ behavior-based clustering, ^72^ psychometric network analysis, ^83^ and SHAP-grounded, LLM-generated justifications for individual risk classifications ^64^ to provide human-understandable risk scores and forensic insights for intervention strategies.

### Lightweight and edge AI: operational efficiency

Operational efficiency is the final barrier to national-scale implementation, particularly in resource-constrained environments. Kuntoro et al., ^1^ Harahap and Ridho, ^2^ and Ghani et al. ^75^ deployed detection or mitigation directly on DNS infrastructure, IDS sensors, or institutional networks, and Erawan et al. ^3^ kept memory use below 20 MB on government servers, indicating that future solutions must run on such infrastructure without disrupting public services. Ma et al. ^76^ showed that a graph-based method using textual content avoids the overhead of visual features and achieved the shortest CPU time among the compared methods, and Kurniawan and Triayudi ^74^ reconstructed and detected defacement attacks with open-source tools (Wazuh and Velociraptor) on cloud-hosted servers. Overall, the included studies point toward systems that are self-learning, cross-platform (Web 2.0 and Web 3.0), and linguistically aware rather than rigid and reactive.

## Discussion

This section synthesizes the findings from the four research questions and discusses the broader implications of the current state of the OGDS. By analyzing 39 primary studies, we identified a clear paradigm shift in detection strategies and the remaining hurdles for real-world implementation.

### The evolution of detection paradigms

The results for RQ1 and RQ2 revealed a transition from static, single-modality detection to dynamic, multimodal, and graph-based architectures. Signature- and keyword-based filtering, such as Suricata rules built from TF-IDF bigram keywords ^2^ and keyword scanning of search-engine results, ^60^ and conventional machine learning, such as SVM optimized by particle swarm optimization ^73^ and Random Forest combined with Isolation Forest, ^75^ remain common in the included studies. However, as documented in our synthesis, modern systems leverage Deep Learning (e.g., CNN, LSTM, and Transformers such as IndoBERT) to capture complex semantic nuances ^47^ ^,^ ^54^ and visual promotional cues. ^50^ ^,^ ^59^

Furthermore, the rise of Graph Neural Networks (GNNs), such as HGNN and TacDroid, ^51^ ^,^ ^53^ signifies a shift from analyzing content to analyzing relationships. This is particularly vital in the Web3 and Blockchain era, where transaction patterns may be harder for operators to disguise than website content. ^91^ ^,^ ^94^

### The adversarial arms race and linguistic barriers

A key finding from RQ3 is the intensifying “arms race” between gambling operators and detection systems. Operators have evolved beyond simple URL obfuscation ^57^ to sophisticated tactics such as “multimodal hiding” (embedding text in distorted images) as shown in Figure 10 ^49^ ^,^ ^50^ and “web defacement” in high-authority government and academic domains (.go.id and.ac.id). ^2^ ^,^ ^74^ Figure 10 illustrates this technique, which we call watermarked advertising images: it obscures textual clues by separating visual signals from semantic indicators of promotional gambling and appears not only on websites but also in social media content.

Screenshots of social media posts in which promotional text is embedded in images, separating visual signals from textual cues. All account names, usernames/handles, profile identifiers, URLs, and other potentially identifying information have been fully obscured to prevent identification.

Our analysis also highlights the critical geographic and linguistic gaps. Although global models are robust, they often struggle with regional slang and dialects. In the Indonesian context, terms like “ *gacor*” or “ *jepe*” are a moving target for static filters. ^47^ ^,^ ^54^ This underscores the need for localized context-aware NLP models that are continuously updated to reflect cultural and linguistic shifts.

### Operational feasibility versus algorithmic complexity

The synthesis of implementation challenges reveals a stark contrast between academic performance and operational reality. Although complex GNN and visual-language models achieve high accuracy, deploying them on low-spec government servers or high-throughput ISP infrastructure remains difficult because of high VRAM and CPU costs. ^76^ Our findings suggest that for national-scale mitigation, lightweight solutions—such as optimized bash scripts ^3^ and DNS RPZ clustering ^1^—are often more sustainable than heavy deep-learning frameworks.

The reviewed studies share a common pipeline. To synthesize the heterogeneous design choices reported in the primary studies, we distilled the OGDS workflow into a unified pipeline ( Figure 11). The workflow begins with diverse modalities, including web text, promotional images, mobile application UI transition graphs, transaction logs, and network traffic packets. These inputs are then processed through detection architectures implemented as convolutional-based (for visual features), attention-based (IndoBERT/transformers), graph-based (HGNN/GCN), and specialized lightweight variants. Depending on the regulatory objective, architectures are applied to a wide range of tasks, such as website detection, mobile app classification, social media and spam filtering, and harm detection. The resulting outputs are ultimately channeled into operational utilities, supporting government-level blocking (e.g., TrustPositif ), ISP real-time mitigation, financial forensics for law enforcement, and early intervention to prevent harm. This unified view highlights that despite methodological diversity, the reviewed studies converge on a common pipeline in which advanced AI serves as a versatile backbone bridging raw digital footprints and actionable regulatory insights.

The reviewed papers showed that nearly half of the included studies (18 of 39) focused on website (15) and mobile application (3) detection, reflecting the global urgency to mitigate the proliferation of illegal platforms. Although this concentration provides strong evidence of the effectiveness of multimodal and graph-based methods in platform identification, it also highlights an underexplored frontier: extending the OGDS to harm detection and psychological behavioral analysis in decentralized (Web 3.0) and high-throughput real-time settings. These areas represent promising directions for future research, particularly given the increasing complexity of adversarial evasion tactics and emergence of blockchain-based gambling ecosystems. Figure 12 summarizes the resulting landscape of OGDS methods and their characteristics.

### Service detection versus harm and behavioral risk profiling

We included behavioral and psychometric studies because the scope of this review (target iv in the OGDS definition) covers gambling-related harm and risk behavior inferred from platform data, and because operators and regulatory bodies draw on the same transaction logs and activity records that support enforcement. ^44^ ^,^ ^56^ ^,^ ^63^ Placing these studies beside service detection shows how far computational methods now extend from infrastructure to users, but it does not make the two tasks equivalent. They differ in their unit of analysis, their reference standard, and the consequences of error. Service detection classifies websites, applications, messages, domains, or transactions whose labels can be checked against observable artefacts such as page content, application behavior, or regulator blocklists, and an error means that a legitimate service is blocked or an illegal one is missed. Harm-oriented studies score or group people or populations against reference standards that are themselves indirect: a list of players previously excluded for problematic gambling, ^56^ self-reported Problem Gambling Severity Index scores, ^63^ labels refined with the model’s own feature thresholds, ^64^ internal clustering indices without an external criterion, ^44^ ^,^ ^45^ or survey scales and model-fit indices; ^46^ ^,^ ^83^ two further studies analyzed population-level forum activity ^84^ and commercial churn ^86^ rather than individual harm. None of the nine studies used a clinician-administered diagnostic assessment as its reference standard, so their metrics measure agreement with each study’s proxy rather than diagnostic accuracy. Stechschulte et al., ^56^ for example, reported an area under the curve of 92.58% but a precision of 2.40%, meaning that roughly one in 42 flagged players belonged to the target group, and Murch et al. ^63^ showed that decision thresholds had to be recalibrated as behavior shifted. A false positive in this setting labels a person rather than a service, with implications for privacy, stigma, and access to support.

Consequently, the high precision and F1-scores reported for website, mobile application, and message detection ( Tables 13–15) should not be read as evidence that harm-oriented models can diagnose gambling disorder, and the harm-domain results ( Table 16) should not be ranked against them. We interpret harm models as risk-screening and triage aids whose outputs require human review and, where an intervention is considered, clinical assessment, and we treat the psychometric studies ^46^ ^,^ ^83^ ^,^ ^84^ as contextual evidence on risk markers rather than as detection systems. Future harm-detection studies should report their reference standard, the prevalence of the target class, and positive predictive value alongside discrimination metrics, and should validate against clinically assessed outcomes where this is ethically feasible.

### Critical appraisal of the evidence base

The included studies scored highly on the quality checklist (mean 8.6 of 10; range 6–10), but the individual checklist items reveal systematic weaknesses that the total score conceals. Twelve of the 39 studies (31%) did not compare their method against a baseline, so their reported gains may reflect the dataset rather than the proposed design. Nine studies (23%) reported no bias control, data validation, scalability, or reliability measures, and seven (18%) did not describe their system architecture in enough detail to reimplement it.

Reproducibility and evaluation realism are also limited. Only 12 studies (31%) released code or a dataset ( Table 19); the remaining 27 evaluated their models on data that other researchers cannot access or re-test. Only six studies reported computational cost, such as memory use, CPU time, or detection latency, even though deployment on government servers and ISP infrastructure is the main use case the field invokes. Evaluation under realistic conditions was rare: in our extraction matrix, HeCGamb ^51^ was the only study to report an open-world evaluation, in which its performance decreased (F1-score 94.1%, AUC 96.8%), and Murch et al. ^63^ were the only authors to test whether model performance changes over time.

The evidence is also geographically concentrated. Eighteen of the 39 studies (46%) came from authors based in China and seven (18%) from Indonesia, so most findings reflect two regulatory environments, their languages, and their platforms. A high score on one national dataset does not show that a model will transfer to another language or market. Taken together, these patterns indicate that the field has demonstrated detection feasibility within individual settings but has not yet shown generalizability, resilience to adaptive operators, or cost-effectiveness at operational scale. Before ranking architectures, shared benchmarks with temporal splits, adversarial test sets, and mandatory reporting of computational cost are needed.

### Limitations of this review

Several limitations should be considered when interpreting this review. (i) Selection bias was mitigated by a predefined search string and multi-stage filtering; however, the search covered four databases, English-language publications, and the period 2021–2025, and database subject-area filters restricted records to computer science. Relevant gray literature and studies indexed under other disciplines (e.g., gambling studies or psychology journals) may therefore have been missed; for example, two scoping reviews of player-level risk-detection models ^104^ ^,^ ^105^ were not retrieved by the search and were identified only during revision ( Table 2). We could not retrieve four potentially relevant reports in full text and excluded them, including multimodal, graph-based, and blockchain detection studies ^69^ ^,^ ^70^ ^,^ ^88^ and a security assessment of Android gambling apps ^81^; we cite them in the Introduction based on their abstracts only. (ii) Several studies cited as background in the Introduction ^5^ ^,^ ^55^ ^,^ ^61^ ^,^ ^65^ passed quality assessment but were excluded at the secondary eligibility stage because they lacked sufficient architectural detail or a reproducible technical evaluation; they inform the context of this review but not its synthesis. (iii) Performance values were extracted as reported by the primary studies, which used different datasets, splits, and metrics; no meta-analysis, risk-of-bias assessment beyond the quality checklist, assessment of reporting bias, or certainty-of-evidence grading was performed, so cross-study comparisons are descriptive. (iv) The findings rest on 39 primary studies, and the rapid evolution of the gambling ecosystem means that new evasion tactics may emerge before they are documented in peer-reviewed literature. (v) The categorization of challenges and methods was reached through iterative consensus among the authors to ensure that the extracted data reflected the intent of the original studies. (vi) The review protocol was defined before the search ( Figure 2) but was not prospectively registered in a public registry such as OSF or PROSPERO, and no separate protocol document was published; its elements are reported in Figure 2 and Tables 2– 5. The secondary eligibility criterion was added after quality assessment (see Inter-rater reliability). (vii) The quality checklist was purpose-built for engineering evaluations and has not been validated as a risk-of-bias instrument. (viii) Inter-rater agreement was quantified for the eligibility decision after quality assessment; agreement statistics were not computed for title and abstract screening or for data extraction, and quality scoring and data extraction were performed by one reviewer and verified by a second rather than duplicated independently, and the reinstated study ^64^ was judged for secondary eligibility jointly rather than independently. (ix) The composition of the corpus, including its concentration in China and Indonesia, reflects the English-language literature indexed by the four databases under the stated filters; it should not be read as the global distribution of detection research or operational systems, which are often documented only in non-English or gray literature. (x) The broad definition of OGDS, which includes harm and risk-behavior detection alongside the detection of illegal services, increases coverage but reduces comparability across domains. Metrics in the harm domain measure agreement with proxy reference standards, such as exclusion lists, self-report screening scores, or internal clustering indices, rather than clinical diagnostic accuracy, and this review did not appraise the clinical validity of those instruments (see “Service detection versus harm and behavioral risk profiling”). Findings should therefore be compared within, rather than across, the six operational domains.

In summary, Figure 12 presents the comprehensive landscape of Online Gambling Detection System methods and their characteristics.

**Figure 12.**
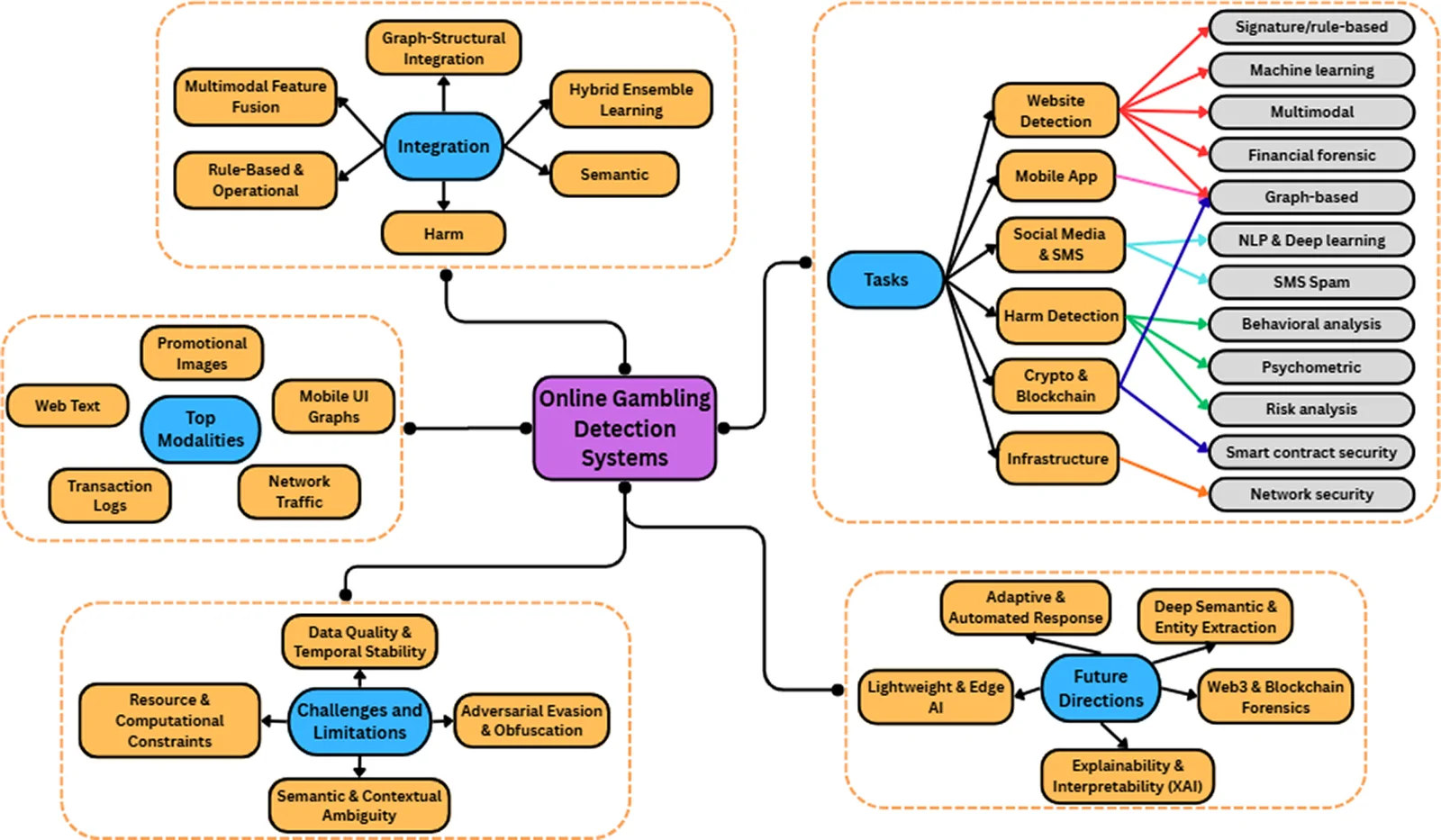
Summary of the online gambling detection systems landscape. Overview linking modalities, integration patterns, domain tasks, challenges and limitations, and future directions identified in this review.

## Conclusions

This study presents an SLR for OGDS, charting the evolution of technology from passive content blocking to an adaptive, intelligent forensic ecosystem. Based on a synthesis of empirical evidence, the study draws four key conclusions that contribute to the understanding of the current cybersecurity landscape.

First, a Detection Paradigm Shift. The analysis of RQ1 and RQ2 confirms a fundamental transition from static rule-based (signature-based) methods to dynamic Deep Learning and Graph-based architectures. Although traditional methods such as IDS Suricata ^2^ and DNS RPZ ^1^ remain relevant for infrastructure-level efficiency, modern methods leveraging Vision-Language models, Graph Neural Networks (GNN), ^52^ and transformers (such as IndoBERT) ^47^ ^,^ ^54^ reported the highest detection scores against complex evasion techniques among the included studies. Integrating multimodal features (text, visual, and behavioral) has been shown to improve detection accuracy to above 98% when distinguishing gambling sites from normal ones using sophisticated cloaking techniques. ^50^ ^,^ ^62^

Second, Operational and Linguistic Gaps exist. Addressing RQ3, this study identified a critical gap between academic performance and real-world feasibility. Although complex models such as GNNs and multimodal fusion achieve high precision, their application to resource-constrained government infrastructure is often impractical because of their high computational requirements. ^76^ Furthermore, semantic challenges remain a major obstacle, particularly in the Indonesian context, where the use of rapidly evolving slang terms (such as “ *gacor*” or “ *jepe*”) and obfuscation techniques on social media platforms often fail to be detected by global language models that are not locally calibrated. ^47^ ^,^ ^54^

Third, The Urgency of Behavioral Detection and Blockchain Forensics. This study highlights the fact that content detection alone is no longer sufficient. The research focus is shifting towards user behavior analysis for harm detection ^44^ ^,^ ^63^ and decentralized infrastructure forensics (blockchain). ^91^ ^,^ ^94^ These findings emphasize that the future of OGDS is not just about blocking URL access, but also about understanding financial transaction patterns and the network structure of malicious actors operating behind the anonymity of cryptocurrencies. Behavioral models of this kind screen users for risk; they do not diagnose gambling disorder.

Fourth, Future Research Directions are needed. In response to RQ4, this study formulated a research roadmap that prioritizes developing adaptive and Autonomous systems. Future researchers should focus on (i) Adaptive Recalibration: Developing models that are resilient to concept drift (changing attack patterns over time) without the need for intensive manual relabeling ^63^; (ii) Lightweight AI: Creating efficient models that can run on low-resource edge devices or government servers without substantially sacrificing accuracy ^3^ ^,^ ^75^; and (iii) Web3 Forensics: Deepening de-anonymization techniques on blockchain networks to track illicit fund flows. ^6^ ^,^ ^92^

Overall, to our knowledge, this SLR provides the first PRISMA-guided cross-platform synthesis restricted to online gambling detection systems. Its primary contributions are a taxonomy that connects input modalities, detection architectures, tasks, and operational uses, and an appraisal showing that much of the reported performance comes from closed datasets, a small number of countries, and evaluations without baselines, temporal validation, or cost reporting. Addressing these gaps, supported by the open data released with this review, is a precondition for comparable benchmarks and for detection systems that can be deployed and defended in regulatory settings.

## Data Availability

Zenodo: Online Gambling Detection Systems: A Systematic Review. https://doi.org/10.5281/zenodo.23175694 ^115^ This project contains the following underlying data:
•
OGDS_SLR_underlying_data.xlsx (Curated dataset prepared for this review, without abstracts, with one worksheet per review stage): ○ README (Search string, search date, and record counts at each screening stage.) ○
01_search_filters (Records identified in Scopus, ScienceDirect, IEEE Xplore, and ACM Digital Library, and the number retained after the year, subject-area, document-type, and language filters: 1,866 records reduced to 273.) ○
02_records_273 (Bibliographic records of the 273 records retained after database filters, with DOI, author keywords, and title/abstract screening outcome.) ○
03_fulltext_97 (The 97 reports sought for retrieval, with the full-text decision and exclusion reason for each.) ○
04_quality_55 (Answers to the 10-item quality checklist, total score, inclusion threshold flag (score ≥ 5), final status, and availability of a public dataset or code for 55 studies.) ○
05_included_39 (Data extraction matrix and reported evaluation metrics for the 39 included studies.) ○ 06_reviewers (Independent include/exclude decisions of Reviewer A and Reviewer B for the 53 studies assessed independently, the joint decision for the reinstated study, the consensus decisions, and the calculation of Cohen’s kappa.) •
Figure 1. tif–Figure 12.tif (Figures 1–12 of this article in TIFF format.) • Figure5_network.zip (term-extraction script, VOSviewer thesaurus file, list of DOIs, per-study term lists, the VOSviewer input network, and the map exported from VOSviewer Online 1.2.4 that were used to generate Figure 5; see the README file in the archive.) We removed abstracts from OGDS_SLR_underlying_data.xlsx and from the Figure 5 archive because publishers hold the copyright; each record is identified by its DOI. Data are available under the terms of the Creative Commons Attribution 4.0 International license (CC-BY 4.0). Zenodo: Online Gambling Detection Systems: A Systematic Review. https://doi.org/10.5281/zenodo.23175694 ^115^ This project contains the following reporting guidelines:
•
OGDS_PRISMA_2020_checklist.docx (Completed PRISMA 2020 checklist indicating where each item is reported.) •
Figure 3. tif (PRISMA 2020 flow diagram of study identification, screening, and inclusion; identical to Figure 3.) Data are available under the terms of the Creative Commons Zero “No rights reserved” data waiver (CC0 1.0 Public domain dedication).
