# Supplementary material for: Online Gambling Detection Systems: A Systematic Review
Source: F1000Res. 2026 Nov 11;15:1763. Originally published 2026 Oct 22. [Version 2] doi: 10.12688/f1000research.191912.2 (PMC13760149; doi:10.12688/f1000research.191912.2)
Supplement: Peer review history

# Reviewer report: Reviewer response for version 2

**DOI:** 10.5256/f1000research.214250.r540747

**Published:** 2026-11-12

**Authors:** Syifaurachman Syifaurachman

**Affiliations:** 1 Universitas Pamulang, Tangerang, Banten, Indonesia

no further concerns, I have reviewed the revised manuscript, and it is well-crafted in its discussion, offering valuable perspectives on the challenges and opportunities within the field of Online Gambling Detection Systems, as well as in its exploration of multimodal approaches.

Are the rationale for, and objectives of, the Systematic Review clearly stated?

Yes

Is the statistical analysis and its interpretation appropriate?

Yes

If this is a Living Systematic Review, is the ‘living’ method appropriate and is the search schedule clearly defined and justified? (‘Living Systematic Review’ or a variation of this term should be included in the title.)

Not applicable

Are sufficient details of the methods and analysis provided to allow replication by others?

Yes

Are the conclusions drawn adequately supported by the results presented in the review?

Yes

Reviewer Expertise:

cybersecurity, machine learning, applied informatics

I confirm that I have read this submission and believe that I have an appropriate level of expertise to confirm that it is of an acceptable scientific standard.

# Reviewer report: Reviewer response for version 2

**DOI:** 10.5256/f1000research.214250.r540746

**Published:** 2026-11-12

**Authors:** Afandi Thohari

**Affiliations:** 1 Politeknik Negeri Semarang, Semarang, Central Java, Indonesia

The author has revised the manuscript according to my comments.

Are the rationale for, and objectives of, the Systematic Review clearly stated?

Yes

Is the statistical analysis and its interpretation appropriate?

Yes

If this is a Living Systematic Review, is the ‘living’ method appropriate and is the search schedule clearly defined and justified? (‘Living Systematic Review’ or a variation of this term should be included in the title.)

Yes

Are sufficient details of the methods and analysis provided to allow replication by others?

Yes

Are the conclusions drawn adequately supported by the results presented in the review?

Yes

Reviewer Expertise:

deep learning, computer vision, multimodal learning

I confirm that I have read this submission and believe that I have an appropriate level of expertise to confirm that it is of an acceptable scientific standard.

# Reviewer report: Reviewer response for version 1

**DOI:** 10.5256/f1000research.211835.r536177

**Published:** 2026-11-03

**Authors:** Afandi Thohari

**Affiliations:** 1 Politeknik Negeri Semarang, Semarang, Central Java, Indonesia

This paper presents a systematic literature review of online gambling detection systems (OGDS), conducted in accordance with the PRISMA 2020 statement. The authors searched IEEE Xplore, the ACM Digital Library, ScienceDirect, and Scopus for studies published between 2021 and 2025, narrowing 1866 records to 39 primary studies through a 10-item quality assessment and independent eligibility judgments by two reviewers (κ = 0.83). The included studies are mapped onto six operational domains, namely website detection, harm detection, social media and SMS, cryptocurrency and blockchain, mobile applications, and network infrastructure, and onto six architectural integration patterns ranging from multimodal and graph-based fusion to rule-based operational systems. Multimodal and graph-based models reported the highest detection scores against content obfuscation, whereas lightweight rule-based approaches were favored in resource-constrained infrastructure. The evidence base, however, remains uneven, as 12 studies lacked a baseline comparison, only 12 released code or data, and only six reported computational cost. The authors identify four recurrent challenges, namely temporal drift, adversarial evasion, semantic ambiguity arising from localized slang and blockchain anonymity, and computational constraints, and propose a research agenda centered on adaptive systems, entity extraction, Web3 forensics, explainable AI, lightweight edge deployment, and shared cross-regional benchmarks. Nevertheless, several aspects of the manuscript could be further improved, as outlined below.

1. The claim that this is the first review of online gambling detection systems is currently supported only by a narrative paragraph in the Related work section. Please add a table comparing this review with similar secondary studies, outlining for each study its scope, the detection tasks it addresses, its primary focus, and how it falls short of the present SLR. The table should include the reviews already cited and the survey studies excluded at full-text screening. This would make explicit how the present review differs from prior work and better justify its novelty claim.
2. The text currently highlights terms such as “detection”, “website”, and “classification” that do not appear in Figure 5, so the description and the figure should be aligned. Please confirm that the keyword co-occurrence network was generated directly from the studies retrieved and included through the reported search, rather than from manually curated or merged terms. Given that the network is based on only 39 studies, its interpretation, as well as the explanations offered for the year-to-year changes in Figure 4, should be presented as indicative rather than confirmatory. To improve readability, please also redraw Figure 5 with a white background instead of the current black one.
3. The manuscript contains many single-sentence paragraphs, particularly at the start of the RQ3 and RQ4 subsections and around figures and tables (e.g., pp. 8, 14, 20, 23, 27–31, 33–34). Please merge these sentences into adjacent paragraphs or develop them into fuller paragraphs to improve the flow and readability of the text.

Are the rationale for, and objectives of, the Systematic Review clearly stated?

Yes

Is the statistical analysis and its interpretation appropriate?

Yes

If this is a Living Systematic Review, is the ‘living’ method appropriate and is the search schedule clearly defined and justified? (‘Living Systematic Review’ or a variation of this term should be included in the title.)

Yes

Are sufficient details of the methods and analysis provided to allow replication by others?

Yes

Are the conclusions drawn adequately supported by the results presented in the review?

Yes

Reviewer Expertise:

deep learning, computer vision, multimodal learning

I confirm that I have read this submission and believe that I have an appropriate level of expertise to confirm that it is of an acceptable scientific standard, however I have significant reservations, as outlined above.

# Response

**Published:** 2026-11-04

**Authors:** Abdul Azzam Ajhari

**Affiliations:** University of Indonesia Faculty of Computer Science, Depok, West Java, Indonesia

We thank both reviewers for their careful reading. Reviewer comments are shown in italics, followed by our response and the corresponding changes. Table and reference numbers refer to Version 2: Tables 2–20 of Version 1 are now Tables 3–21 because we added a new Table 2 and five references.

 Reviewer 2

***Comment 1.*** *Add a table comparing this review with similar secondary studies (scope, detection tasks, primary focus, and how each falls short), including the reviews already cited and the survey studies excluded at full-text screening, to justify the claim that this is the first review.*

**Response. **We added Table 2 to the Related work. It compares this review with twelve secondary studies: the nine reviews cited in Version 1, including the three secondary studies excluded at full-text screening (Musa et al., Gibson et al., and Kuss et al.), and three reviews identified during revision, namely two scoping reviews of player-level risk-detection models (Ghaharian et al., 2023; Marionneau et al., 2025) and a survey of AI-based detection of illicit web ecosystems published after our search date (Hwang and Min, 2026). For each study, the table reports the design, scope or corpus, the OGDS domains covered, the primary focus, the gap relative to this review, and the screening stage at which it was excluded.

**Correction. **While preparing the table, we re-examined the eight reports excluded at full text as “survey studies”. Only three were secondary studies; the other five were primary questionnaire, case, or design studies without a detection method or measurable evaluation metrics, and the label had conflated questionnaire surveys with literature surveys. We corrected the category in the Methods (secondary studies, n = 3; primary studies without a detection method or measurable evaluation metrics, n = 5), in Figure 3, and in the underlying data (sheet 03_fulltext_97). The number of full-text exclusions (38) and the set of 39 included studies are unchanged.

**Novelty statement. **We narrowed the novelty statement to “the first PRISMA-guided systematic review restricted to online gambling detection systems that jointly cover website and mobile detection, social media promotion, infrastructure-level evasion, harm detection, and blockchain forensics, and that appraises how the reported performance was obtained”, made the corresponding change in the Conclusions, and noted in Limitation (i) that the two scoping reviews were not retrieved by our search.

***Comment 2.*** *The text highlights “detection”, “website”, and “classification”, which do not appear in Figure 5; confirm that the network was generated directly from the included studies rather than from manually curated or merged terms; present the interpretation of Figures 4 and 5 as indicative; and redraw Figure 5 on a white background.*

**Response. **The Version 1 network was produced in VOSviewer Online and contained combined labels (e.g., “Blockchain & Cryptocurrency”) whose derivation was not documented, so we could not confirm that it had been generated without manual curation. We therefore rebuilt Figure 5 from the titles and abstracts of the 39 included studies. We extracted noun phrases using part-of-speech tagging, reduced plural nouns to the singular, and counted each term once per study; we merged only spelling variants and abbreviations, and removed generic research terms using a VOSviewer thesaurus file that lists every change. The 35 terms that occur in at least three studies and their 284 co-occurrence links were then loaded into VOSviewer Online 1.2.4, which normalized the links by association strength and computed the layout and the clustering at its default parameters (attraction 2, repulsion 1, resolution 1), yielding five clusters. We exported the map and figure using VOSviewer’s Save and Screenshot functions on a light background; we did not manually edit any term, link, position, or cluster. The extraction script, thesaurus, input network, and exported VOSviewer map are deposited with the underlying data, and repeated runs reproduce identical coordinates and clusters.

**Text alignment and interpretation. **The text now discusses only terms that appear in the figure; “detection” and “website” are now nodes, and “classification” is no longer mentioned because it did not reach the threshold as a standalone term. We rewrote the interpretation of both figures to reflect this. For Figure 4, the text now states that the annual counts refer to included studies rather than total research output, that the 2023 decrease is a difference of three studies, that external developments are context rather than tested explanations, and that the 2025 count may be affected by indexing lag; we also removed the unsupported contrast between conference and journal papers. For Figure 5, the text states that the clusters indicate emphasis within this corpus of 39 studies and do not establish the thematic structure of the wider field.

***Comment 3.*** *Merge the many single-sentence paragraphs, especially at the start of the RQ3 and RQ4 subsections and around figures and tables.*

**Response. **We merged single-sentence paragraphs throughout. In particular, the RQ3 and RQ4 introductions now each form one paragraph; the one-sentence lead-ins and bulleted items of the four RQ3 and five RQ4 subsections were rewritten as continuous prose; the paragraphs that introduce and interpret each performance table were combined into one paragraph per domain that precedes the table; and the review-protocol list, the inter-rater agreement passage, the Figure 2, Figure 10, and Figure 12 sentences, and the research-question lead-in were merged with adjacent text. Apart from the three lead-ins to displayed equations and the figure legends, the remaining one-sentence paragraphs are list items and statements in the Data availability and declaration sections.

 Other changes

 We replaced Figure 7 with a bar chart of the country counts reported in the text; corrected the attribution of the 3.65% class-imbalance figure, which comes from Stechschulte et al. rather than Ma et al., in the text and in Table 20; replaced Figure 3 to show the corrected exclusion categories; added to Table 16 the note on reference standards requested in the first round; replaced the word “significant” where no statistical test was involved; unified the DOIs in the Data availability section; restyled two subsection headings that had been set as body text; and removed publisher abstracts from the deposited workbook. An “Amendments from Version 1” section summarizes these changes.

# Reviewer report: Reviewer response for version 1

**DOI:** 10.5256/f1000research.211835.r533098

**Published:** 2026-10-26

**Authors:** Syifaurachman Syifaurachman

**Affiliations:** 1 Universitas Pamulang, Tangerang, Banten, Indonesia

The authors are required to clearly articulate the rationale for including psychometric behavioral analysis within the framework of an engineering detection system. Kindly incorporate a specific paragraph in the Discussion or Limitations that clarifies the distinction between cybersecurity-oriented service detection and clinical/behavioral harm profiles, to making certain that readers do not confuse the precision of infrastructural detection with the effectiveness of clinical diagnostics.

Are the rationale for, and objectives of, the Systematic Review clearly stated?

Yes

Is the statistical analysis and its interpretation appropriate?

Yes

If this is a Living Systematic Review, is the ‘living’ method appropriate and is the search schedule clearly defined and justified? (‘Living Systematic Review’ or a variation of this term should be included in the title.)

Not applicable

Are sufficient details of the methods and analysis provided to allow replication by others?

Yes

Are the conclusions drawn adequately supported by the results presented in the review?

Yes

Reviewer Expertise:

cybersecurity, machine learning, applied informatics

I confirm that I have read this submission and believe that I have an appropriate level of expertise to confirm that it is of an acceptable scientific standard.

# Response

**Published:** 2026-11-04

**Authors:** Abdul Azzam Ajhari

**Affiliations:** University of Indonesia Faculty of Computer Science, Depok, West Java, Indonesia

We thank both reviewers for their careful reading. Reviewer comments are shown in italics, followed by our response and the corresponding changes. Table and reference numbers refer to Version 2: Tables 2–20 of Version 1 are now Tables 3–21 because we added a new Table 2 and five references.

 Reviewer 1

*The authors are required to clearly articulate the rationale for including psychometric behavioral analysis within the framework of an engineering detection system. Kindly incorporate a specific paragraph in the Discussion or Limitations that clarifies the distinction between cybersecurity-oriented service detection and clinical/behavioral harm profiles, to making certain that readers do not confuse the precision of infrastructural detection with the effectiveness of clinical diagnostics.*

**Response. **We added the Discussion subsection “Service detection versus harm and behavioral risk profiling”. It explains that behavioral and psychometric studies were included because the review scope (target iv of the OGDS definition) covers gambling-related harm and risk behavior inferred from the same platform data that operators and regulators use, and it distinguishes the two tasks by unit of analysis, reference standard, and consequence of error. The subsection lists the reference standard used by each of the nine harm-domain studies (exclusion lists, self-reported PGSI scores, model-refined labels, internal clustering indices, survey scales, forum activity, and churn), notes that none used a clinician-administered diagnostic assessment, and illustrates the gap between discrimination and precision with Stechschulte et al. (AUC 92.58%, precision 2.40%). It states explicitly that the precision reported for website, mobile application, and message detection (Tables 13–15) should not be read as evidence of diagnostic effectiveness and that the harm-domain results (Table 16) should not be ranked against them.

**Further changes. **We also (i) added the rationale for target (iv) to the Methods; (ii) added a sentence to the Harm detection subsection of the Results stating that these studies score individuals against proxy reference standards; (iii) added a note to Table 16 stating that its metrics are not measures of clinical diagnostic accuracy; (iv) expanded Limitation (x); (v) added a sentence to the Conclusions stating that behavioral models screen users for risk and do not diagnose gambling disorder; and (vi) replaced diagnostic wording (“pathological”, “addiction”) in the Introduction, Related work, Results, and Table 1 with “potentially problematic gambling” or “gambling-related harm”, and revised the description of Ling et al. to match the findings reported in that study.
